# Improved Bioavailability and Bioaccessibility of Lutein and Isoflavones in Cultured Cells In Vitro through Interaction with Ginger, Curcuma and Black Pepper Extracts

**DOI:** 10.3390/antiox11101917

**Published:** 2022-09-27

**Authors:** Bernhard Blank-Landeshammer, Gerald Klanert, Lisa Mitter, Sophia Turisser, Nicolas Nusser, Alice König, Marcus Iken, Julian Weghuber

**Affiliations:** 1 Center of Excellence Food Technology and Nutrition, University of Applied Sciences Upper Austria, Stelzhamerstraße 23, 4600 Wels, Austria; 2 FFoQSI GmbH—Austrian Competence Centre for Feed and Food Quality, Safety and Innovation, Technopark 1D, 3430 Tulln, Austria; 3 PM International AG, Schengen, 5445 Luxembourg, Luxembourg

**Keywords:** lutein, isoflavones, bioavailability, bioenhancer, P-glycoprotein, CaCo-2

## Abstract

Intestinal absorption is intrinsically low for lipophilic micronutrients and phytochemicals. Plant extracts acting as bioavailability enhancers can complement for this deficiency by modulation of both, physicochemical and biochemical parameters, in the absorption process. However, these interactions often are limited to specific conditions and the mechanisms and potential synergisms are poorly understood. In this work, we used a human intestinal cell line to characterize the impact of extracts from *C. longa* (curcuma), *Z. officinale* (ginger) and *P.nigrum* (black pepper) on uptake and transport rates of the xanthophylls lutein and zeaxanthin as well as soy isoflavones measured by HPLC-DAD. We found a significant increase in the uptake of lutein in the presence of curcuma extract and enhanced isoflavone transport rates mediated by curcuma and ginger extracts. Combinations of the plant extracts did not lead to any additional increase in uptake or transport rates. By investigation of mixed micelle incorporation efficiency, we could dismiss changes in bioaccessibility as a potential enhancing mechanism in our experimental setup. We further conducted a rhodamine 123 efflux assay and discovered inhibition of P-glycoproteins by the ginger and black pepper extracts, highlighting a plausible route of action leading to increased isoflavone bioavailability.

## 1. Introduction

Dietary phytochemicals are a large group of plant metabolites such as polyphenols, carotenoids, saponins, terpenes, glycosides alkaloids or anthocyanins found in plant-based foods. Many of these compounds are known to exert beneficial health effects upon consumption and some are essential for human development and well-being [1,2].

The xanthophyll lutein and its stereoisomer zeaxanthin are plant pigments present in various leafy green vegetables, flowers and colored fruits. Upon consumption, they are partly transformed to meso-zeaxanthin and mainly found in the retina and referred to as macula pigment, where they serve as photoprotectors and enhance visual performance. This protective effect is mediated by filtering high-energy blue light and by acting as an antioxidant to scavenge reactive oxygen species [3,4]. It was shown that lutein reduces the progression of age-related macular degeneration (AMD) by suppression of inflammation and reduction of local oxidative stress [5].

Isoflavones, a subset of the large group of phenolic plant compounds, are mainly found in soybeans and other legumes. These flavonoids have been shown to possess anti-inflammatory, antimicrobial, antioxidant and anticancer properties [6,7]. Furthermore, due to their structural resemblance to estrogens, isoflavones are considered to act as endocrine disruptors. By blocking the binding of more potent estrogens, isoflavones such as genistein are thought to reduce the risk of developing hormone-related cancers such as breast, ovary and prostate cancer as well as to reduce menopausal symptoms [8,9].

Both of these phytochemicals are characterized by their relatively low bioavailability, i.e., only a small portion of the substances is being effectively absorbed to reach the site of action in the body without being modified or excreted. Bioavailability is an attribute governed by a multitude of factors and conditions. Hydrophobic compounds such as carotenes and xanthophylls need to be solubilized in the gut lumen to be efficiently taken up, both passively and by active transport via scavenger receptor class B type 1 (SR-BI) and cluster of differentiation 36 (CD36). Other phytochemicals such as isoflavones are passively diffusing through the membrane of intestinal epithelial cells, but are targets of phase-I and -II metabolism and thus are largely modified, degraded or exported before reaching their potential target sites [2].

Bioenhancers as a concept stem from traditional Indian medicine and ayurvedic therapy, thought to act as enhancing agents on the uptake and efficacy of co-ingested substances. Numerous plant extracts have been screened for their bioenhancing properties, with black pepper and its main active constituent piperine—a part of the ayurvedic formulation of ‘Trikatu’—being the first and probably best characterized. Uptake of curcumin, a constituent of the rhizome of *Curcuma longa*, can be increased 10-fold when co-ingested with piperine. Furthermore, the bioavailability of a number of drugs can similarly be enhanced, but the effect is not of general nature [10]. Inhibition of the efflux transporters P-glycoprotein (P-gp) as well as breast-cancer resistance protein (BCRP) and multi-drug resistance protein 2 (MDR2) were found to be the strategic points for interference [11].

However, prediction of intestinal nutrient and drug absorption efficiency in general and determination of synergistic or antagonistic interactions of food and drug components is challenging. Even though demonstrating invaluable advantages, most animal models suffer from poor predictability of human intestinal drug absorption. This is due to vast differences in the expression profiles of drug transporters and metabolizing enzymes [12,13,14]. Consequently, immortalized human intestinal cell lines such as CaCo-2 and LS-180 have been established as convenient tools to study drug absorption and their interplay with transporting and metabolizing enzymes [15]. They have been successfully utilized by our group, for instance, to investigate the uptake of micellated algae oil [16].

In this work, we implemented the CaCo-2 model to characterize the potential bioenhancing effect of a *C. longa* extract (CuE) complexed with γ-cyclodextrin (CD) to improve its solubility and a *Zingiber officinale* (ginger) extract (GiE) on the uptake and transport of particular isoflavone and lutein formulations as well as potential synergistic effects with a *P. nigrum* (black pepper) extract (BPE). Glucuronide and sulfate conjugates of the isoflavones were monitored to identify their possible influence on phase-II metabolism enzyme classes UGT and SULT. Furthermore, to additionally characterize the possible mode of action of the extracts, we determined their interplay on the incorporation efficiency of lutein and isoflavones into mixed micelles and performed transporter assays to specifically pinpoint their effects on the efflux transporter P-gp.

## 2. Materials and Methods

### 2.1. Chemicals and Reagents

Lutein and Zeaxanthin (in the following denoted simply as lutein) in micellated formulation as well as the corresponding oil were obtained from Lipoid (Ludwigshafen, Germany), while lutein in powdered formulation was received from Divi’s Laboratories Europe AG (Basel, Switzerland). Analytical (all-E)-lutein and meso-zeaxanthin standards were purchased from Sigma Aldrich (St. Louis, LO, USA). Isoflavone in micellated formulation was obtained from Lipoid (Ludwigshafen, Germany), while the bulk isoflavone was retrieved from Frutarom (Londerzeel, Belgium). Analytical standards for the aglycones glycitein, daidzein and genistein as well as for the glycosylated forms (glycitin, daidzin, genistin) were purchased from Extrasynthese (Lyon, France). Curcuma extract complexed with γ-cyclodextrin was obtained from Wacker Chemie (Munich, Germany), while pure curcuma extract was purchased from Sigma Aldrich (St. Louis, LO, USA) and analytical curcuminoid standards (curcumin, demethoxycurcumin, bisdemethoxycurcumin) were purchased from Extrasynthese (Lyon, France). Black pepper extract was provided by Sabinsa Europe GmbH (Langen, Germany) and ginger extract was obtained from Vivatis Pharma (Hamburg, Germany).

### 2.2. CaCo-2 Cell Culture

Human Caco-2 cells were purchased from DSMZ (Braunschweig, Germany). The cells were maintained in Earl’s Minimal Essential Medium (MEM) with non-essential amino acids supplemented with 10% fetal bovine serum (FBS) and 100 U/mL penicillin/100 μg/mL streptomycin (all PAN-Biotech, Aidenbach, Germany) and grown at 37 °C in a humidified atmosphere (≥95%) with 5% CO_2_. For cell differentiation, cells were seeded in 96-well plates or trans-well inserts and grown overnight. On the next day, the growth medium was aspirated and replaced by Entero-STIM Intestinal Epithelium Differentiation Medium supplemented with 0.1% MITO^+^ Serum Extender (Corning, Wiesbaden, Germany). The differentiation medium was refreshed on day two after seeding. Three days after seeding, the differentiation process was completed, observed by the formation of domes under the microscope [17].

For uptake and transport studies, the target substances were dissolved in fasted state simulated intestinal fluid (FaSSIF) medium [18] and applied to the cells as indicated in Section 2.4 and Section 2.5. FaSSIF was prepared as described by Ilardia-Arana et al. [19] with a final concentration of 3 mM for taurocholic acid (Sigma Aldrich, St. Louis, LO, USA) and 0.75 mM for L-α-lecithin (Merck KGaA, Darmstadt, Germany) in Hanks’ Balanced Salt Solution (HBSS) adjusted to a pH of 6.5.

### 2.3. Cell Viability Assay

For cell viability assays, Caco-2 cells were seeded in black 96-well plates (Greiner-Bio One, Kremsmünster, Austria) at 2 × 10^4^ cells per well, grown overnight under standard conditions and differentiated as described above. Then, the cells were treated with the respective extracts in full growth medium for 4 h. CellTiter-Glo (CTG) luminescent cell viability Assay kit (Promega Corporation, Madison, WI, USA) was used according to the manufacturer’s instructions. Resazurin-based in vitro toxicology assay kit (Sigma Aldrich, St. Louis, LO, USA) was used according to the manufacturer’s instructions. The extract was aspirated, and the cells were lysed with 100 µL of CellTiter-Glo reagent and incubated for 10 min. Subsequently, the ATP luminescent signal was determined with a microplate reader (POLARstar Omega, BMG LABTECH, Ortenberg, Germany).

### 2.4. Lutein and Curcumin Uptake Studies

HPLC measurements (see Section 2.8) were conducted prior to the experiment to normalize the total xantophyll content in the different formulations and concentrations showing no significantly adverse effects on cell viability were dissolved in FaSSIF medium. Caco-2 cells were seeded in 15 cm dishes and differentiated as described above. After removal of differentiation medium, lutein formulations and respective plant extracts in FaSSIF medium were applied to the cells and incubated for a period of 4 h. The supernatant was collected for subsequent HPLC analysis and cells were washed twice with FaSSIF medium, twice with PBS and finally detached from the dishes using a cell scraper and resuspended in PBS. After centrifugation and removal of PBS, xantophyll esters were hydrolyzed by saponification according to Toomey et al. [18]. Cell pellets were resuspended in 0.75 M ethanolic KOH and butylated hydroxytoluene (BHT, Sigma Aldrich, St. Louis, LO, USA) was added at a final concentration of 0.1% (*w*/*v*). Pellets were broken up using an ultrasonic lancet (20 pulses á 1s) and an aliquot was taken for protein determination by Micro-Bradford assay (BioRad, Hercules, CA, USA) according to the manufacturer’s protocol. Saponification was carried out for 4 h on a thermoshaker set to 40 °C and 500 rpm shaking in the dark. Xantophyll extraction was then carried out by addition of n-hexane/diethyl ether (1/1, *v*/*v*), and saturated NaCl. The organic phase was carefully transferred to a new vial and the extraction procedure was repeated twice. The united organic phases were dried completely under a vacuum and resuspended in 100 µL acetonitrile for HPLC measurement.

### 2.5. Isoflavone Transport Studies

For transport studies, cells were seeded at 5.5 × 10^5^ cells/trans-well insert (ThinCert, 0.336 cm^2^, 0.4 μm pore diameter; Greiner-Bio One, Kremsmünster, Austria) and differentiated using Entero-STIM Intestinal Epithelium Differentiation Medium (Corning, Wiesbaden, Germany) supplemented with 1% penicillin/streptomycin and 0.1% MITO+ Serum Extender (Corning). Monolayer integrity was assessed by transepithelial electrical resistance (TEER) measurement with a Millicell-ERS-2 Voltohmmeter (Merck, Darmstadt, Germany) as described previously [20]. For experiments, differentiated cells were washed twice with HEPES buffer. To mimic intestinal microbial digestion, isoflavones were first incubated with β-glucosidase (2 U/mL) in 50 mM MES buffer (pH 5.5) at 37 °C for 2 h and then diluted in FaSSIF medium with addition of the respective plant extracts. An aliquot was taken for later analysis to determine the isoflavone concentration prior to incubation. After removal of the differentiation medium from the apical side of the cell monolayer, FaSSIF medium was added and incubation was continued for 4 h. Afterwards, apical and basolateral medium were collected for HPLC analysis. An aliquot from each compartment was diluted with acetonitrile and directly measured to determine the level of isoflavone aglycones. A second aliquot was incubated with β-glucuronidase (200 U/mL glucuronidase and 1 U/mL sulfatase activity) at 37 °C for 1 h prior to dilution with acetonitrile and HPLC measurement to determine the portion of phase-II metabolites in the media.

### 2.6. Determination of Incorporation of Lutein and Isoflavones into Mixed Micelles

Incorporation efficiency into mixed micelles was determined as described previously with slight modifications [21]. Stock solutions of the lipid components were prepared in chloroform-methanol (2/1, *v*/*v*) and aliquots were transferred to glass tubes to accomplish the following concentrations in the final mixture: 0.1 mM cholesterol, 0.3 mM monoolein, 0.5 mM oleic acid and 0.2 mM phosphatidylcholine. Concentrations of lutein and isoflavone products were calculated to resemble physiological doses in a fasted state [22], corresponding to 67 µg/mL or 118 µM total xantophylls and 50 µg/mL or 113 µM of total isoflavones, while plant extracts were added in the same ratio as previously applied in CaCo-2 uptake and transport studies. After solvent evaporation under nitrogen, dried residues were solubilized in 10 mL of 5 mM sodium taurocholate (in 0.9% NaCl) by ultrasonication for 10 min. After filtration through 0.2 µM syringe filters, incorporation efficiency was determined by HPLC measurement as described above.

### 2.7. Rhodamine Efflux Assay

For P-gp activity assays, Caco-2 cells were seeded in black 96-well plates at 2 × 10^4^ cells per well and grown for 48 h under standard conditions. After media removal, cells were washed with HBSS and incubated for 30 min with 10 µM rhodamine 123 in HBSS. Cells were then put on ice and washed twice with ice-cold HBSS before adding plant extracts or verapamil dissolved in FaSSIF. After incubation for 60 min at standard conditions, cells were put on ice again and washed with ice-cold DPBS (PAN Biotech GmbH, Aidenbach, Germany). Cells were then lysed with 100 µl/well RIPA lysis buffer (150 mM NaCl, 1% Triton X-100, 0.5% sodium dodecyl sulfate (SDS) and 50 mM Tris-HCl (pH 8.0)) and incubated at room temperature for 30 min. Fluorescence intensity of intracellular rhodamine 123 was measured at 485 nm excitation wavelength and 520 nm emission wavelength using a microplate reader (POLARstar^®^ Omega, BMG LABTECH, Ortenberg, Germany).

### 2.8. HPLC Analysis

Target substances were analyzed by HPLC-DAD and HPLC-FLD using an external standard calibration. Separation was carried out with an Ultimate 3000 HPLC System equipped with an Accucore C18 column (150 × 3 mm, 2.6 μm particle size, both Thermo Fisher Scientific, Bremen, Germany) and 0.1% formic acid (FA) in water as solvent A and 0.1% FA in acetonitrile as solvent B. For separation of xantophylls, a binary gradient was employed starting at 80% solvent B, then risen to 94% B in 4 min, maintained at 94% B for 10 min, then decreased back to 80% B in 1 min and finally equilibrated for 10 min. The column compartment was maintained at 25 °C and signals were obtained at a wavelength of 445 nm. For isoflavone separation, the gradient was started at 12% B and maintained for 5 min, then risen to 38% B in 8 min, 70% B in 5 min and 80% B in 1 min, which was held constant for 1.5 min, followed by a decrease back to 12% B and a 10 min equilibration. The column temperature was held at 40 °C and UV signals were recorded at wavelengths 249 and 260 nm. For detection of curcuminoids, the same gradient and column parameters were used, but detection was carried out with a fluorescence detector with excitation and emission wavelengths set to 432 and 535 nm respectively.

### 2.9. Statistics

Data analysis was performed using R statistical software (version 3.6.2, The R project for statistical computing, Vienna, Austria) and one-way ANOVA was carried out when only two conditions were compared, while two-way ANOVA followed by post-hoc Tukey’s HSD for correction of multiple testing was employed when comparing multiple conditions. Significant differences are denoted as * (*p*-value ≤ 0.05), ** (≤0.01) and *** (≤0.001).

## 3. Results

### 3.1. Quantitation of Bioactive Lead Compounds in Lutein and Isoflavone Extracts

To compare the impact of different formulations on the cellular uptake of lutein and isoflavone extracts, the concentration of the active compounds was determined to enable normalized administration. For *C. longa* extracts, the concentration of the three main curcuminoids was determined by HPLC-FLD (Table 1, Supplementary Figure S1). In both cases they showed comparable ratios and no discrimination by the CD-complexation process was apparent. Therefore, the total curcuminoid content was used for further normalization.

Similarly, the content of xantophylls in three different lutein formulations was determined by HPLC-DAD (Table 2, Supplementary Figure S2). While in the powdered formulation complexed with modified starch, all xantophylls were present in their free form and could be directly measured, xantophyll esters in the oil and the micellated product had to be subjected to saponification prior to analysis. Conditions were carefully optimized with regard to extraction efficiency and stability. No significant degradation of either lutein or zeaxanthin was observable with the chosen saponification conditions (Supplementary Figure S3). In all probes, (all-E)-lutein was identified as the main geometrical isomer, making up 79 to 87% of the total quantified xantophylls. By comparison of retention time and absorbance spectra to literature [23], the presence of (13-Z)-lutein was confirmed as a minor constituent. Due to the absence of pure analytical standards, (all-E)-lutein was used to indirectly quantify the content of this geometrical isomer. To correct for the differences in isomer composition, the total quantified xantophyll content was used to normalize the applied amount in cell culture experiments.

Finally, the concentration of micellated and powder isoflavone products was quantified by HPLC-DAD (Table 3, Supplementary Figure S4). In both cases, isoflavones were mainly present in their glycosylated form, making up 95% of the respective isoflavone pool, with the aglycones present in trace amounts.

Moreover, CuE, GiE and BPE were analyzed for the presence of potentially interfering traces of isoflavones or lutein. The observed signal was clearly above the lower limit of detection of the method (Supplementary Figure S5).

### 3.2. Curcuma Extract Increases Lutein Uptake in Intestinal Cells

Cellular bioavailability and potential interactions of lutein and isoflavone products with the applied bioenhancer extracts were determined in human CaCo-2 cells. Unless otherwise stated, the studies were conducted with differentiated cells to mimic enterocytes of the human small intestine [24,25]. To better simulate the physiological conditions of the human gastrointestinal tract, biorelevant media are commonly applied in pharmaceutical and clinical research [26]. All extracts were therefore dissolved in fasted state simulated intestinal fluid (FaSSIF) prior to administration. Initial concentration ranges for the application were retrieved from literature and tested for their potential cytotoxicity (Supplementary Figure S6). For final uptake and transport studies, concentrations with no significant impact on cellular viability under the applied conditions were chosen for each extract.

**Figure 1:**
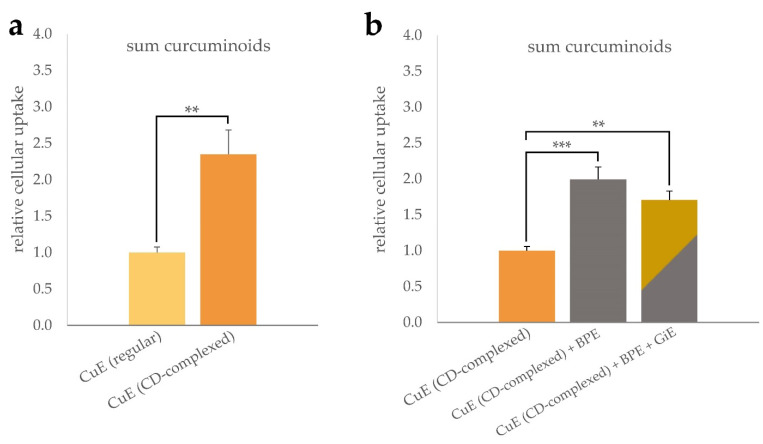
Uptake of curcuminoids in differentiated CaCo-2 cells. Cells were incubated for 4 h with regular or CD-complexed curcuma extracts at a final concentration of 2.95 µg/mL of total curcuminoids. For comparison of the formulations, the cellular curcuminoid content was determined and, after normalization to the protein content, the relative cellular uptake was calculated with reference to the regular extract (**a**). Influence of BPE and GiE at concentrations of 6 µg/mL and 59 µg/mL on the curcuminoid uptake were normalized to CuE (**b**). In both cases, control experiments without addition of CuE were performed. Error bars represent mean ± SEM (*n* > 6). Statistically significant differences are denoted as ** (*p* ≤ 0.01) and *** (≤0.001). Corresponding raw data are available in Supplementary Tables S1 and S2.

As shown in Figure 1a, incubation with CD-complexed curcuma extract (CuE) resulted in a more than two-fold increase of the intracellular curcuminoid concentration compared to the unformulated control. Co-incubation of the CuE with black pepper extract (BPE) at a concentration of 6 µg/mL led to a further statistically significant increase of the cellular curcuminoid uptake (Figure 1b). Addition of ginger extract (GiE) at a concentration of 59 µg/mL did not further increase the intracellular curcuminoid-concentration significantly after the 4 h incubation period.

**Figure 2:**
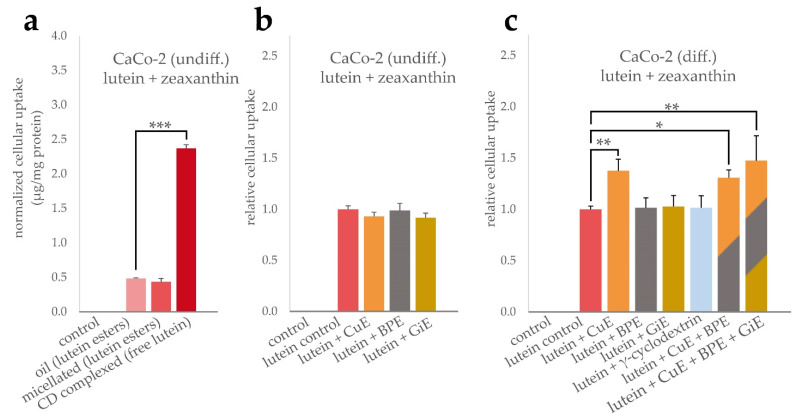
Uptake of lutein and zeaxanthin in CaCo-2 cells. To compare the uptake efficiency of the lutein formulations, cells were incubated for 4 h with lutein oil, micellated lutein or CD-complexed lutein at a final concentration of 14.7 µg/mL lutein + zeaxanthin. Cellular uptake was quantified by HPLC after saponification and normalized to the total protein content (**a**). Influence of BPE, CuE and GiE at concentrations of 6 µg/mL, 14.7 µg/mL and 59 µg/mL, respectively, on the cellular uptake of lutein were determined both in undifferentiated (**b**) and differentiated cells (**c**). Combinations of the extracts and addition of γ-cyclodextrin were studied in differentiated cells (**c**). In all cases, control experiments were performed without the addition of lutein and bioenhancer extracts. Error bars represent mean ± SEM (*n* > 6). Statistically significant differences are denoted as * (*p* ≤ 0.05), ** (≤0.01) and *** (≤0.001). Corresponding raw data are available in Supplementary Table S3–S5.

Similarly, the cellular uptake of lutein formulations and potential interactions with BPE, GiE and CuE were determined. As indicated in Figure 2a, the cellular bioavailability of the free lutein powder was significantly higher compared to both, micellated and unformulated lutein esters, by a factor of 5 with an average of 2.37 µg total xantophylls per mg cellular protein content. However, in our experimental setup, no significant difference in the bioavailability of the latter two lutein formulations was found. The average cellular uptake was 0.48 µg/mg protein for the oil and 0.43 µg/mg protein for the micellated formulation. Due to the higher bioavailability in these initial experiments, the lutein powder formulation was used to further investigate the interaction with the bioenhancer extracts. In undifferentiated CaCo-2 cells, no significant changes in the cellular lutein + zeaxanthin concentration was found when co-incubated with any of the bioenhancer extracts (Figure 2b). In contrast, in differentiated CaCo-2 cells the uptake of lutein was significantly increased by 38% when CuE was applied simultaneously, but no changes were detectable for BPE or GiE (Figure 2c). As BPE significantly increased the cellular uptake of curcuminoids, it was further tested whether this augmented intracellular concentration also had an impact on the uptake of lutein. As shown in Figure 2c, simultaneous incubation of lutein powder with CuE and BPE or with CuE, BPE and GiE did not enhance the uptake of lutein to an extent beyond the effect of CuE alone. Additionally, we tested the effect of γ-cyclodextrin to rule out an enhanced lutein uptake mediated by this reagent, as cyclodextrins are known to alter membrane cholesterol composition and can be internalized by CaCo-2 cells [27]. As expected, no significant change in the controls was detectable.

### 3.3. CuE and GiE Enhance Isoflavone Transport by CaCo-2 Cells

**Figure 3:**
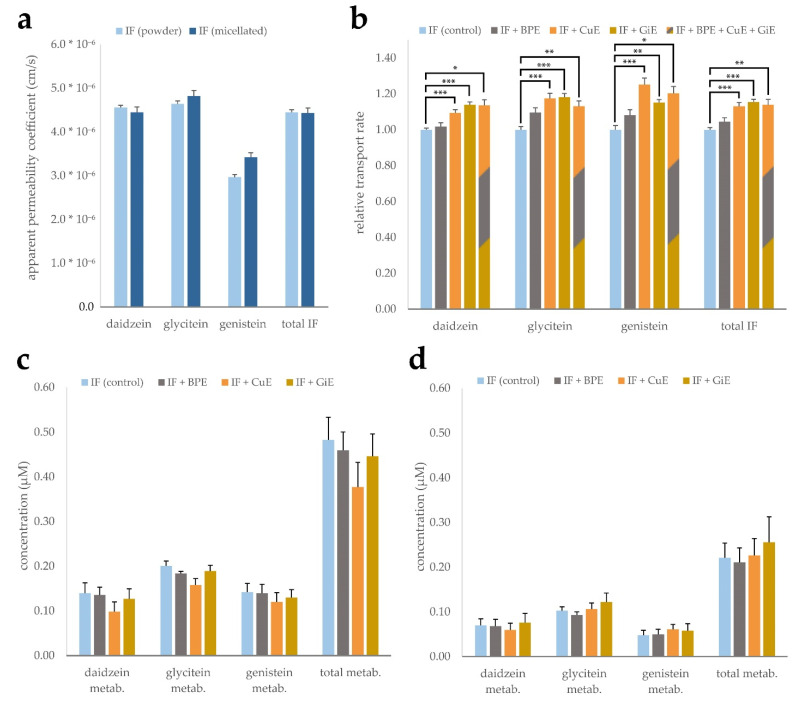
Transport of the isoflavones (IF) daidzein, glycitein and genistein across a CaCo-2 monolayer. Micellated and CD-complexed isoflavone products were normalized to a total content of 8.5 µg/mL and applied after β-glucosidase digestion. Apparent permeability coefficients were calculated after determination of the concentrations in the basolateral compartment after 4 h incubation and are depicted for each aglycone individually as well as for the sum of isoflavones (total IF) (**a**). Effects of BPE, GiE and CuE were evaluated in both formulations individually and are depicted jointly by calculation of the transport rate relative to the respective control (**b**). Isoflavone metabolite concentrations in the apical (**c**) and basolateral (**d**) compartments based on determination by β-glucuronidase digestion. Error bars represent mean ± SEM (*n* = 6). Statistically significant differences are denoted as * (*p* ≤ 0.05), ** (≤0.01) and *** (≤0.001). Corresponding raw data are available in Supplementary Table S6–S8.

To study the effects of powder and micellar formulation, as well as the potential impact of CuE, BPE and GiE extracts on isoflavone bioavailability, CaCo-2 monolayer trans-well assays were conducted. Both isoflavone products mainly consist of isoflavone glycosides, which in vivo are deconjugated prior to intestinal absorption mainly by the gut microflora [28,29]. To account for this, all isoflavone products were digested with β-glucosidase prior to application to convert them to their aglycone form. Digested products were added to the apical medium, and after 4 h of incubation the concentrations in the apical and basolateral compartments were measured. These values were used to calculate the apparent permeability coefficient (P_app_), i.e., the permeability rate divided by the initial concentration and the surface area of the monolayer. While daidzein and glycitein showed similar transport rates of 4.45 to 4.82 × 10^−5^ cm/s, the P_app_ of genistein was considerably lower with 2.97 to 3.42 × 10^−5^ cm/s. However, no significant differences were found between the micellar and CD-complexed isoflavone formulations (Figure 3a).

Influences of the individual bioenhancer extracts as well as in combination on the transport rate of the isoflavones were investigated for both formulations. Experiments were conducted with both micellated and powder formulations and the transport rates were normalized and combined to balance out minor differences between the formulations and better resolve the impact of the bioenhancer extracts (Figure 3b). Co-incubation with Curcuma extract led to an increase of the P_app_ ranging from 10 to 25% for the individual isoflavone aglycones and 13% for the sum of all isoflavones when compared to the control group without extract addition. Similarly, ginger extract enhanced the transport rate by 14 to 18% in case of the individual substances and by 15% for the sum of isoflavones. Addition of black pepper extract did not lead to a significant increase of the P_app_ of any of the isoflavones. Combined application of all three extracts did not lead to an augmentation of the transport rate surpassing the effect of any of the individual extracts.

As isoflavones are known to undergo phase-II metabolism, differences in the occurrence of glucuronate and sulfate conjugates might indicate interaction of the plant extracts with SULT und UGT enzymes. Since no corresponding standards were available for quantification, we chose to indirectly measure metabolite concentrations by digestion of the apical and basolateral media with β-glucuronidase. Presence of the metabolites can then be deduced by quantification of the isoflavone aglycones prior and after digestion. Calculated concentrations of the metabolites in the control samples ranged from 0.14 to 0.20 µM in the apical compartment and 0.07 to 0.1 µM in the basolateral compartment. The relative share of metabolites with respect to the total amount was lowest for daidzein with 2.4% in the apical compartment and 10.8% in the basolateral compartment. Genistein showed the highest relative metabolization rate, with the metabolites accounting for 9.6% of the total genistein pool in the apical compartment and 38.5% in the basolateral compartment. Addition of the individual plant extracts did not lead to significant changes in the metabolite concentrations in both compartments (Figure 3c,d). Of note, samples co-incubated with CuE showed a significant overall reduction of metabolites in the apical compartment in individual experiments, while the combined reduction of 22% was not statistically significant due to high inter-experimental variation.

### 3.4. Impact of Bioenhancer Extracts on Mixed Micelle Incorpartion Efficiency of Lutein and Isoflavones

Intestinal absorption of lipophilic micronutrients is largely governed by their potential to be solubilized and incorporated into mixed micelles [30]. The composition of mixed micelles influences their solubilization capacities and it has been shown that some phytochemicals are able to influence mixed micelle incorporation efficiency [31]. We therefore investigated the impact of the bioenhancer plant extracts CuE, BPE and GiE on the mixed micelle incorporation efficiency of the lutein and isoflavone formulations, as it may contribute to increased cellular uptake and transport rates, respectively.

**Figure 4:**
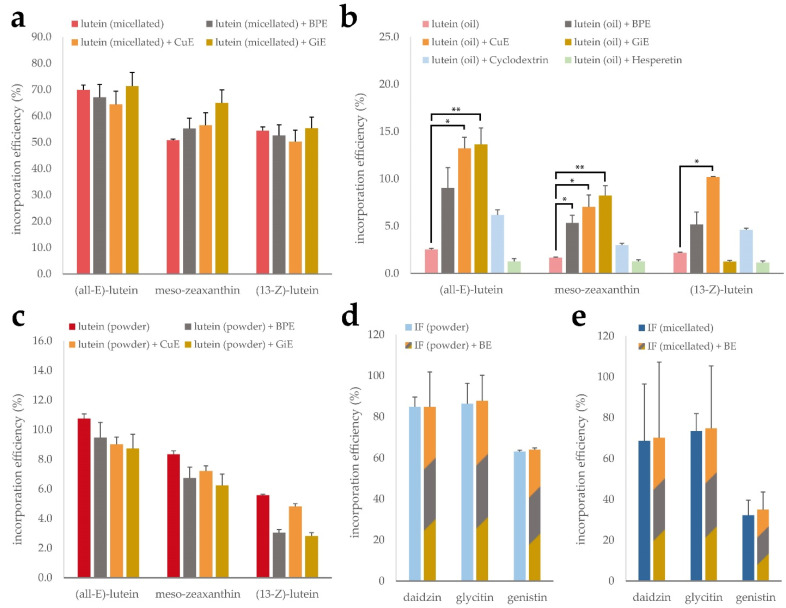
Mixed micelle incorporation efficiency of micellated lutein (**a**), lutein oil (**b**) and free lutein powder (**c**) in combination with CuE, BPE and GiE, respectively, as well as CD-complexed and micellated isoflavones (IF) with a mixture of the extracts (**d**,**e**). Error bars represent mean ± SEM (*n* = 3). Statistically significant differences are denoted as * (*p* ≤ 0.05) and ** (≤0.01).

As expected, none of the tested extracts led to a significant difference in the incorporation efficiency of (all-E)-lutein, (13-Z)-lutein or meso-zeaxanthin in the micellated lutein product, as the synthetic micelles remain intact in this process. This is reflected in the high overall incorporation efficiency of 50 to 70% (Figure 4a). However, for the unformulated lutein oil, the incorporation efficiencies of the individual isomers only ranged from 1.7 to 2.5%. The addition of CuE during the micellization process led to a 5-fold increase of the incorporated lutein fraction throughout all isomers. Similarly, GiE addition was accompanied by an increase of the (all-E)-lutein incorporation efficiency to almost 14%. Interestingly, this effect was not observable for the (13-Z) isomer. Incorporation efficiencies of the lutein oil in the presence of BPE resulted in a non-significant increase (Figure 4b). Nie et al. have recently reported an increased incorporation efficiency of β-carotene in the presence of the citrus flavonoids hesperetin and hesperidin [31], however we could not confirm this effect using our experimental conditions. Free lutein showed intermediate incorporation efficiency and did not benefit from the addition of any of the plant extracts (Figure 4c).

To investigate the impact of the bioenhancer extracts on solubilization efficiency of the isoflavones, both, the CD-complexed and the micellated product, were subjected to the micellization procedure with and without the addition of a mixture of BPE, CuE and GiE (Figure 4d,e). For both formulations, the basic incorporation efficiency was highest for dadizin and glycitin compared to genistin, ranging from 63 to 86% in the CD-complexed product and from 32 to 74% in the micellated formulation. However, no beneficial effect of the bioenhancer extract mixture was detected on any of the isoflavones.

### 3.5. Rhodamine Efflux Assay

**Figure 5:**
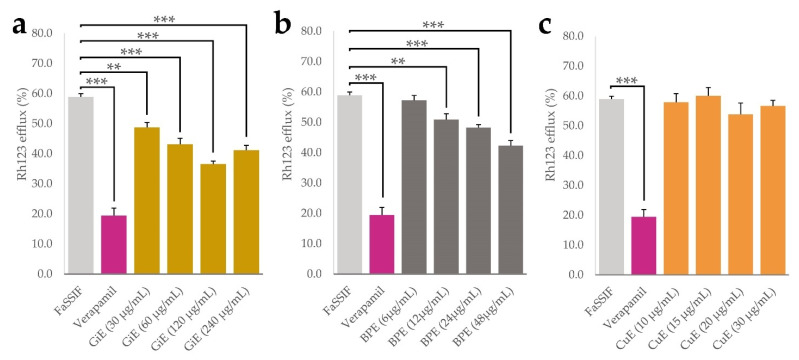
Results of the rhodamine 123 efflux assay, indicated as a percentage of the control without Rh123 efflux. After 30 min accumulation with Rh123 (10 µM), GiE (**a**), BPE (**b**) and CuE (**c**) were applied in 4 different concentrations with the lowest one representing their respective concentrations in transport and uptake tests, while the concentration of verapamil was 50 µM. FaSSIF medium was used as a negative control. After 1 h of incubation, intracellular Rh123 concentrations were determined. Error bars represent mean ± SEM (*n* = 9–12). Statistically significant differences are denoted as ** (*p* ≤0.01) and *** (≤0.001).

Aside from physicochemical processes such as solubilization and micellization, bioavailability of plant phytochemicals is influenced by numerous cellular processes taking place at the intestinal epithelium. P-glycoprotein 1 (P-gp) is an important transport protein located on the luminal side of intestinal epithelial cells and mediates the efflux of numerous xenobiotics. It was shown that inhibition of P-gp might be one of the main mechanisms that leads to enhanced curcumin uptake in numerous model systems [32,33]. We therefore conducted experiments with CaCo-2 cells using the P-gp substrate rhodamine 123 (Rh123) as a molecular probe and the drug verapamil as a positive control for P-gp inhibition [34,35]. By measuring the intracellular Rh123 accumulation, effects of the plant extracts in four different concentrations on its efflux were quantified, with lower efflux indicating potential P-gp inhibition. As expected, after incubation with verapamil, Rh123 efflux was significantly lower than in the negative control only treated with FaSSIF medium, with an efflux rate of 18% compared to 60% in the negative control. For BPE, the lowest applied concentration of 6 µg/mL did not significantly decrease Rh123 efflux, but a dose-dependent effect was found in the range between 12 and 48 µg/mL with a minimal efflux of 48% at the highest concentration. GiE significantly decreased Rh123 efflux in all applied concentrations, with a dose-dependent effect between 30 and 120 µg/mL and a minimal efflux of 40%. Application of CuE did not lead to significant changes in Rh123 efflux at any of the applied concentrations. (Figure 5a–c).

## 4. Discussion

Intestinal bioavailability of phytochemicals is governed by both physicochemical and biochemical mechanisms and interactions between individual substances that are difficult to predict [36]. In this study, we investigated the uptake and transport of several plant-based food supplements in different formulations using human intestinal cell models and determined their interactions with known and putative bioenhancers. The term—a contraction of ‘bioavailability enhancer’—was first coined in 1979 referring to the positive effects of piperine on drug bioavailability, while the historic concept is found in Ayurvedic medicine [10]. We found that extracts of *C. longa* and *Z. officinale* increased the cellular uptake of lutein and the transport rate of soy isoflavones, respectively. By performing further molecular assays on their effects on micellization and interaction with transport proteins we were able to narrow down the potential mechanisms that mediate these enhancing effects.

Bioavailability of curcumin extracted from *C. longa* has long been known to benefit from combined administration with *P. nigrum* extract [32]. Complexation of curcumin with γ-cyclodextrin on the other hand has been shown in clinical trials to increase plasma concentration of curcumin and its metabolites by almost 40 times compared to a bulk formulation [37]. In our experimental setup, we were able to confirm the beneficial effect of γ-cyclodextrin complexation, albeit to a much smaller extent. This can be explained by the multitude of additional processes and factors such as digestibility, intestinal motility, hepatic metabolism and many more that are insufficiently addressed by in vitro cell culture experiments. However, our study did not only replicate the effect of the solubility on cellular curcumin uptake, but also indicated the additional increase of the intracellular curcumin concentration through an apparent interaction of piperine with P-gp. This finding enabled us to better validate the CaCo-2 cell model and finally identify phytochemicals that modulate the bioavailability of curcumin.

Comparison of lutein uptake in undifferentiated CaCo-2 cells showed a five-times higher apparent bioavailability for the powder formulation than the micellated or bulk lutein product, both of which contain esterified lutein in contrast to the free molecule in the powder product. Intestinal uptake of xantophylls is not fully understood yet, but occur both by passive diffusion or actively mediated by scavenger receptor class B type 1 (SR-B1), cluster determinant 36 (CD36) and Nieman-Pick C1 Like1 (NPC1L1) [38]. In vivo, xantophylls are mainly taken up in their free form after ester hydrolysis by intestinal lipases, a process which is known to be insufficiently represented in CaCo-2 cell models [39]. Bioenhancer extracts where therefore tested with the powder formulation as it was thought to best reproduce these uptake mechanisms in vitro. Interestingly, we found a beneficial effect of CuE on lutein uptake, however it was not possible to elevate this effect by co-incubation with BPE, which per se enhances intracellular curcumin levels. Furthermore, the increase in lutein uptake was not observable in undifferentiated CaCo-2 cells. This finding indicates the enhancing effect to be mediated by interaction with a molecular target only expressed in differentiated cells. Chen et al. found that curcumin treatment led to upregulation of CD36 and ABCA1 in M1 macrophages, leading to increased cholesterol uptake [40]. This route can be ruled out, as CaCo-2 cells do not express CD36 [41], hinting at possible interactions of curcumin with SR-B1, the expression of which has been shown to be differentiation-dependent in CaCo2-cells [42]. Mixed micelle formation can likely be excluded as a possible explanation for the CuE effect, as we found no impact on the incorporation efficiency of the bioenhancer extracts on the lutein powder. However, the extracts considerably improved the incorporation efficiency of the bulk lutein oil, with CuE leading to a five-fold increase of lutein in the micellar fraction. This difference is most likely caused by the higher lipophilicity of lutein esters which seem to benefit from the interaction with the phenolic extract compounds.

While we did not observe any differences in the transport rate between the micellated and non-micellated isoflavone products, both CuE and GiE led to a significant increase in basolateral isoflavone concentration in our CaCo-2 monolayer transport assays independent of the product formulation. This effect might be mediated by (i) increased uptake through passive diffusion, (ii) reduced efflux at the apical side of the monolayer or (iii) reduced phase-II metabolism of the isoflavones. To verify the latter, we indirectly quantified isoflavone metabolites in the apical and basolateral compartments by β-glucuronidase digestion which exhibits both glucuronidase and sulfatase activity. GiE-treated samples showed no difference in metabolite concentration compared to the control in neither of the compartments, while overall metabolite concentration was reduced in CuE-treated samples on the apical side. Isoflavones are mainly metabolized by UGT1A and UGT2B, with only a minor contribution of SULT-mediated metabolism [43]. As no reductions in metabolite concentration in the basolateral compartment was found, reduced efflux of the metabolites mediated by CuE interaction with BCRP or MRP2 might be causative for these differences. P-gp is an unlikely candidate for glucuronide transport because of its negatively charged binding pocket [44]. This is in accordance with our findings of BPE and GiE both inhibiting P-gp without showing any significant effect on metabolite concentration in any of the compartments. In agreement with Fang et al., who identified daidzein and glycitein, but not genistein as P-gp substrates, we found the strongest increase in transport rate for the former two in the presence of GiE [45]. We therefore conclude that inhibition of P-gp mediated efflux of isoflavone aglycones might be causative for the enhanced transport rate we observed. Despite the seemingly different enhancement mechanism of CuE, combined treatment with GiE or BPE did not reveal synergistic effects on the isoflavone transport rate in any of the combinations tried. This lack of synergism might be caused by mutual inhibition of the active extract components, but more research is needed to identify the underlying mechanisms.

## 5. Conclusions

In this work, the interaction of three bioenhancer extracts with the target compounds lutein and soy isoflavones provided in different formulations were investigated in a human intestinal cell model. Beneficial effects on the uptake and transport rate were observed for curcuma and ginger extracts. By performance of additional efflux transport assays and micellization studies, a potential mechanism of action was identified that could explain the observed enhancing effect on isoflavone transport.

## Figures and Tables

**Table 1:** Content of the main curcuminoids in regular and CD-complexed curcuma extracts.

	Curcuma Extract (Regular)	Curcuma Extract (CD Complexed)
curcumin (µg/mg)	404.8 ± 11.1 (78.2%)	168.7 ± 1.2 (81.4%)
desmethoxycurcumin (µg/mg)	92.0 ± 3.3 (17.8%)	31.9 ± 0.69 (15.4%)
bisdemethoxycurcumin (µg/mg)	21.0 ± 0.43 (4.0%)	6.6 ± 0.05 (3.2%)
total (µg/mg)	517.7 ± 14.4	207.2 ± 1.9

**Table 2:** Xantophyll content of unformulated lutein, micellated lutein and lutein powder products as determined by HPLC-DAD.

	Lutein (Oil)	Lutein (Micellated)	Lutein (Powder)
(all-E)-lutein (µg/mg)	266.7 ± 22.8	7.2 ± 0.5	65.1 ± 6.3
meso-zeaxanthin (µg/mg)	23.2 ± 0.4	0.3 ± 0.04	5.5 ± 0.2
(13-Z)-lutein (µg/mg)	44.3 ± 1.8	1.5 ± 0.2	3.7 ± 0.1
total (µg/mg)	334.3 ± 24.3	9.1 ± 0.7	74.3 ± 6.2

**Table 3:** Content of isoflavone glycosides and aglycones in micellated and CD-complexed formulations.

	Isoflavones (Micellated)	Isoflavones (Powder)
daidzin (µg/mg)	9.6 ± 0.3	265.7 ± 6.7
glycitin (µg/mg)	3.4 ± 0.2	135.5 ± 4.4
genisitin (µg/mg)	1.7 ± 0.05	35.5 ± 1.2
daidzein (µg/mg)	0.34 ± 0.04	12.8 ± 1.0
glycitein (µg/mg)	0.14 ± 0.03	4.9 ± 0.5
genistein (µg/mg)	0.09 ± 0.02	3.7 ± 0.6
total (µg/mg)	15.3 ± 0.7	458.2 ± 21

## Data Availability

The data presented in this study are available in the main text and in the Supplementary information.

## Supplementary Materials

The following supporting information can be downloaded at: https://www.mdpi.com/article/10.3390/antiox11101917/s1, Figure S1: HPLC-FLD chromatograms of γ-cyclodextrin complexed and bulk curcuma extracts; Figure S2: HPLC-DAD chromatograms of bulk oil, powder and micellated formulation of lutein + zeaxanthin; Figure S3: Optimization of lutein ester saponification conditions; Figure S4: HPLC-DAD chromatograms of micellated and powder formulation of soy isoflavones; Figure S5: HPLC-DAD chromatograms of bioenhancer extracts measured with the isoflavone and lutein methods; Figure S6: Results of cell viability assays of the bioenhancer extracts; Table S1: Raw data for uptake experiments of curcuma formulations in differentiated CaCo-2 cells; Table S2: Raw data for uptake experiments of CD-complexed curcuma with bioenhancer extracts in differentiated CaCo-2 cells; Table S3: Raw data for uptake experiments of lutein formulations in undifferentiated CaCo-2 cells; Table S4: Raw data for uptake experiments of lutein formulations with bioenhancer extracts in undifferentiated CaCo-2 cells; Table S5: Raw data for uptake experiments of lutein formulations with bioenhancer extracts in differentiated CaCo-2 cells; Table S6: Raw data for transport experiments of isoflavone formulations in differentiated CaCo-2 cells; Table S7: Raw data of transport experiments of isoflavone formulations with bioenhancer extracts in differentiated CaCo-2 cells; Table S8: Raw data of metabolite concentrations in transport experiments of isoflavones with bioenhancer extracts in differentiated CaCo-2 cells

## References

[1] Krzyzanowska J., Czubacka A., Oleszek W. (2010). Dietary phytochemicals and human health. Adv. Exp. Med. Biol..

[2] Neilson A.P., Goodrich K.M., Ferruzzi M.G. (2017). Bioavailability and Metabolism of Bioactive Compounds from Foods. Nutrition in the Prevention and Treatment of Disease.

[3] Phelan D., Prado-Cabrero A., Nolan J.M. (2018). Analysis of Lutein, Zeaxanthin, and Meso-Zeaxanthin in the Organs of Carotenoid-Supplemented Chickens. Foods.

[4] Alves-Rodrigues A., Shao A. (2004). The science behind lutein. Toxicol. Lett..

[5] Kamoshita M., Toda E., Osada H., Narimatsu T., Kobayashi S., Tsubota K., Ozawa Y. (2016). Lutein acts via multiple antioxidant pathways in the photo-stressed retina. Sci. Rep..

[6] Yu J., Bi X., Yu B., Chen D. (2016). Isoflavones: Anti-Inflammatory Benefit and Possible Caveats. Nutrients.

[7] Ruiz-Larrea M.B., Mohan A.R., Paganga G., Miller N.J., Bolwell G.P., Rice-Evans C.A. (1997). Antioxidant activity of phytoestrogenic isoflavones. Free Radic. Res..

[8] Jiang H., Fan J., Cheng L., Hu P., Liu R. (2018). The anticancer activity of genistein is increased in estrogen receptor beta 1-positive breast cancer cells. OncoTargets Ther..

[9] Thangavel P., Puga-Olguín A., Rodríguez-Landa J.F., Zepeda R.C. (2019). Genistein as Potential Therapeutic Candidate for Menopausal Symptoms and Other Related Diseases. Molecules.

[10] Atal N., Bedi K.L. (2010). Bioenhancers: Revolutionary concept to market. J. Ayurveda Integr. Med..

[11] Bi X., Yuan Z., Qu B., Zhou H., Liu Z., Xie Y. (2019). Piperine enhances the bioavailability of silybin via inhibition of efflux transporters BCRP and MRP2. Phytomedicine.

[12] Cao X., Gibbs S.T., Fang L., Miller H.A., Landowski C.P., Shin H.-C., Lennernas H., Zhong Y., Amidon G.L., Yu L.X. (2006). Why is it challenging to predict intestinal drug absorption and oral bioavailability in human using rat model. Pharm. Res..

[13] Chu X., Bleasby K., Evers R. (2013). Species differences in drug transporters and implications for translating preclinical findings to humans. Expert Opin. Drug Metab. Toxicol..

[14] Kim H.-R., Park S.-W., Cho H.-J., Chae K.-A., Sung J.-M., Kim J.-S., Landowski C.P., Sun D., Abd El-Aty A.M., Amidon G.L. (2007). Comparative gene expression profiles of intestinal transporters in mice, rats and humans. Pharmacol. Res..

[15] Hubatsch I., Ragnarsson E.G.E., Artursson P. (2007). Determination of drug permeability and prediction of drug absorption in CaCO_2_ monolayers. Nat. Protoc..

[16] Röhrl C., Stübl F., Maier M., Schwarzinger B., Schwarzinger C., Pitsch J., Lanzerstorfer P., Iken M., Weghuber J. (2020). Increased Cellular Uptake of Polyunsaturated Fatty Acids and Phytosterols from Natural Micellar Oil. Nutrients.

[17] Marziano M., Tonello S., Cantù E., Abate G., Vezzoli M., Rungratanawanich W., Serpelloni M., Lopomo N.F., Memo M., Sardini E. (2019). Monitoring CaCO_2_ to enterocyte-like cells differentiation by means of electric impedance analysis on printed sensors. Biochim. Biophys. Acta Gen. Subj..

[18] Klein S. (2010). The use of biorelevant dissolution media to forecast the in vivo performance of a drug. AAPS J..

[19] Ilardia-Arana D., Kristensen H.G., Müllertz A. (2006). Biorelevant dissolution media: Aggregation of amphiphiles and solubility of estradiol. J. Pharm. Sci..

[20] Ollinger N., Neuhauser C., Schwarzinger B., Wallner M., Schwarzinger C., Blank-Landeshammer B., Hager R., Sadova N., Drotarova I., Mathmann K. (2022). Anti-Hyperglycemic Effects of Oils and Extracts Derived from Sea Buckthorn—A Comprehensive Analysis Utilizing In Vitro and In Vivo Models. Mol. Nutr. Food Res..

[21] Homan R., Hamelehle K.L. (1998). Phospholipase A2 relieves phosphatidylcholine inhibition of micellar cholesterol absorption and transport by human intestinal cell line CaCO_2_. J. Lipid Res..

[22] Schiller C., Fröhlich C.-P., Giessmann T., Siegmund W., Mönnikes H., Hosten N., Weitschies W. (2005). Intestinal fluid volumes and transit of dosage forms as assessed by magnetic resonance imaging. Aliment. Pharmacol. Ther..

[23] Khachik F., Englert G., Daitch C.E., Beecher G.R., Tonucci L.H., Lusby W.R. (1992). Isolation and structural elucidation of the geometrical isomers of lutein and zeaxanthin in extracts from human plasma. J. Chromatogr. B Biomed. Sci. Appl..

[24] Levy E., Mehran M., Seidman E. (1995). CaCO_2_ cells as a model for intestinal lipoprotein synthesis and secretion. FASEB J..

[25] Müller U., Stübl F., Schwarzinger B., Sandner G., Iken M., Himmelsbach M., Schwarzinger C., Ollinger N., Stadlbauer V., Höglinger O. (2018). In Vitro and In Vivo Inhibition of Intestinal Glucose Transport by Guava (*Psidium guajava*) Extracts. Mol. Nutr. Food Res..

[26] Boni J.E., Brickl R.S., Dressman J., Pfefferle M.L. (2009). Instant FaSSIF and FeSSIF—Biorelevance Meets Practicality. Dissolution Technol..

[27] Rusznyák Á., Malanga M., Fenyvesi É., Szente L., Váradi J., Bácskay I., Vecsernyés M., Vasvári G., Haimhoffer Á., Fehér P. (2021). Investigation of the Cellular Effects of β-Cyclodextrin Derivatives on CaCO_2_ Intestinal Epithelial Cells. Pharmaceutics.

[28] Islam M.A., Punt A., Spenkelink B., Murk A.J., van Rolaf Leeuwen F.X., Rietjens I.M.C.M. (2014). Conversion of major soy isoflavone glucosides and aglycones in in vitro intestinal models. Mol. Nutr. Food Res..

[29] Setchell K.D., Brown N.M., Desai P., Zimmer-Nechemias L., Wolfe B.E., Brashear W.T., Kirschner A.S., Cassidy A., Heubi J.E. (2001). Bioavailability of pure isoflavones in healthy humans and analysis of commercial soy isoflavone supplements. J. Nutr..

[30] Borel P. (2003). Factors affecting intestinal absorption of highly lipophilic food microconstituents (fat-soluble vitamins, carotenoids and phytosterols). Clin. Chem. Lab. Med..

[31] Nie M., Zhang Z., Liu C., Li D., Huang W., Liu C., Jiang N. (2019). Hesperetin and Hesperidin Improved β-Carotene Incorporation Efficiency, Intestinal Cell Uptake, and Retinoid Concentrations in Tissues. J. Agric. Food Chem..

[32] Shoba G., Joy D., Joseph T., Majeed M., Rajendran R., Srinivas P.S. (1998). Influence of piperine on the pharmacokinetics of curcumin in animals and human volunteers. Planta Med..

[33] Singh D.V., Godbole M.M., Misra K. (2013). A plausible explanation for enhanced bioavailability of P-gp substrates in presence of piperine: Simulation for next generation of P-gp inhibitors. J. Mol. Model..

[34] Jouan E., Le Vée M., Mayati A., Denizot C., Parmentier Y., Fardel O. (2016). Evaluation of P-Glycoprotein Inhibitory Potential Using a Rhodamine 123 Accumulation Assay. Pharmaceutics.

[35] Forster S., Thumser A.E., Hood S.R., Plant N. (2012). Characterization of rhodamine-123 as a tracer dye for use in in vitro drug transport assays. PLoS ONE.

[36] Phan M.A.T., Paterson J., Bucknall M., Arcot J. (2018). Interactions between phytochemicals from fruits and vegetables: Effects on bioactivities and bioavailability. Crit. Rev. Food Sci. Nutr..

[37] Hundshammer C., Schön C., Kimura M., Furune T., Terao K., Elgeti D., Mohr R. (2021). Enhanced metabolic bioavailability of tetrahydrocurcumin after oral supplementation of a γ-cyclodextrin curcumin complex. J. Funct. Foods.

[38] Kopec R.E., Failla M.L. (2018). Recent advances in the bioaccessibility and bioavailability of carotenoids and effects of other dietary lipophiles. J. Food Compos. Anal..

[39] Chitchumroonchokchai C., Failla M.L. (2006). Hydrolysis of zeaxanthin esters by carboxyl ester lipase during digestion facilitates micellarization and uptake of the xanthophyll by CaCO_2_ human intestinal cells. J. Nutr..

[40] Chen F.-Y., Zhou J., Guo N., Ma W.-G., Huang X., Wang H., Yuan Z.-Y. (2015). Curcumin retunes cholesterol transport homeostasis and inflammation response in M1 macrophage to prevent atherosclerosis. Biochem. Biophys. Res. Commun..

[41] Werder M., Han C.H., Wehrli E., Bimmler D., Schulthess G., Hauser H. (2001). Role of scavenger receptors SR-BI and CD36 in selective sterol uptake in the small intestine. Biochemistry.

[42] Cai S.F., Kirby R.J., Howles P.N., Hui D.Y. (2001). Differentiation-dependent expression and localization of the class B type I scavenger receptor in intestine. J. Lipid Res..

[43] Jiang W., Hu M. (2012). Mutual interactions between flavonoids and enzymatic and transporter elements responsible for flavonoid disposition via phase II metabolic pathways. RSC Adv..

[44] Järvinen E., Deng F., Kiander W., Sinokki A., Kidron H., Sjöstedt N. (2021). The Role of Uptake and Efflux Transporters in the Disposition of Glucuronide and Sulfate Conjugates. Front. Pharmacol..

[45] Fang Y., Cao W., Liang F., Xia M., Pan S., Xu X. (2019). Structure affinity relationship and docking studies of flavonoids as substrates of multidrug-resistant associated protein 2 (MRP2) in MDCK/MRP2 cells. Food Chem..

