# Supplementary material for: Improved Bioavailability and Bioaccessibility of Lutein and Isoflavones in Cultured Cells In Vitro through Interaction with Ginger, Curcuma and Black Pepper Extracts
Source: Antioxidants (Basel). 2022 Sep 27;11(10):1917. doi: 10.3390/antiox11101917 (PMC9598320; doi:10.3390/antiox11101917)
Supplement: Supplementary file 1 [file antioxidants-11-01917-s001.zip › antioxidants-1903656-supplementary.pdf]

antioxidants

MDPI

Supplementary Materials: Improved Bioavailability and Bioaccessibility of Lutein and Isoflavones in Cultured Cells In Vitro through Interaction with Ginger, Curcuma and Black Pepper Extracts

Bernhard Blank-Landeshammer [1,2], Gerald Klanert [1,2], Lisa Mitter [2], Sophia Turisser [1], Nicolas Nusser [1], Alice König [1,2], Marcus Iken [3] and Julian Weghuber [1,2,*]

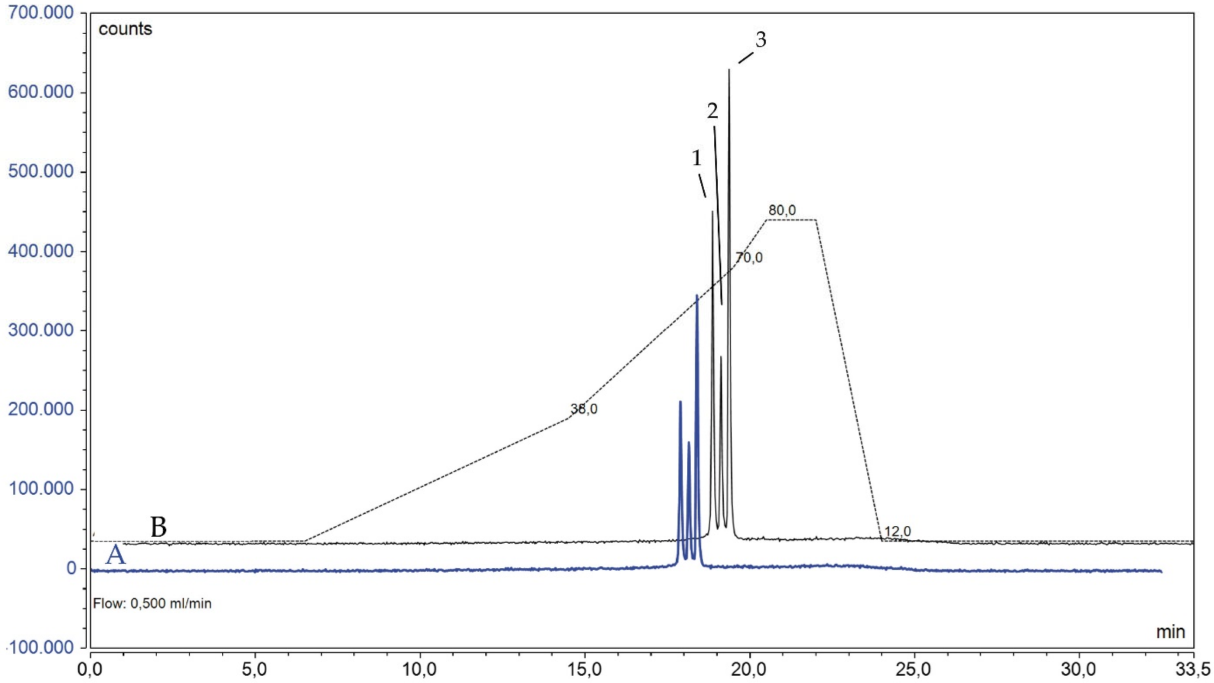

Figure S1. HPLC-FLD chromatograms of γ-cyclodextrin complexed and bulk curcuma extracts. Extinction and emission wavelengths were set to 432 and 535 nm respectively, bulk curcumin extract was dissolved in acetonitrile to a concentration of 3 µg/mL (A), γ-cyclodextrin complexed was dissolved to 12 µg/mL (B). Peak 1 (R_t = 17.89 min) represents bisdemethoxycurcumin, peak 2 (R_t = 18.15 min) represents desmethoxycurcumin and peak 3 (R_t = 18.39 min) represents curcumin.

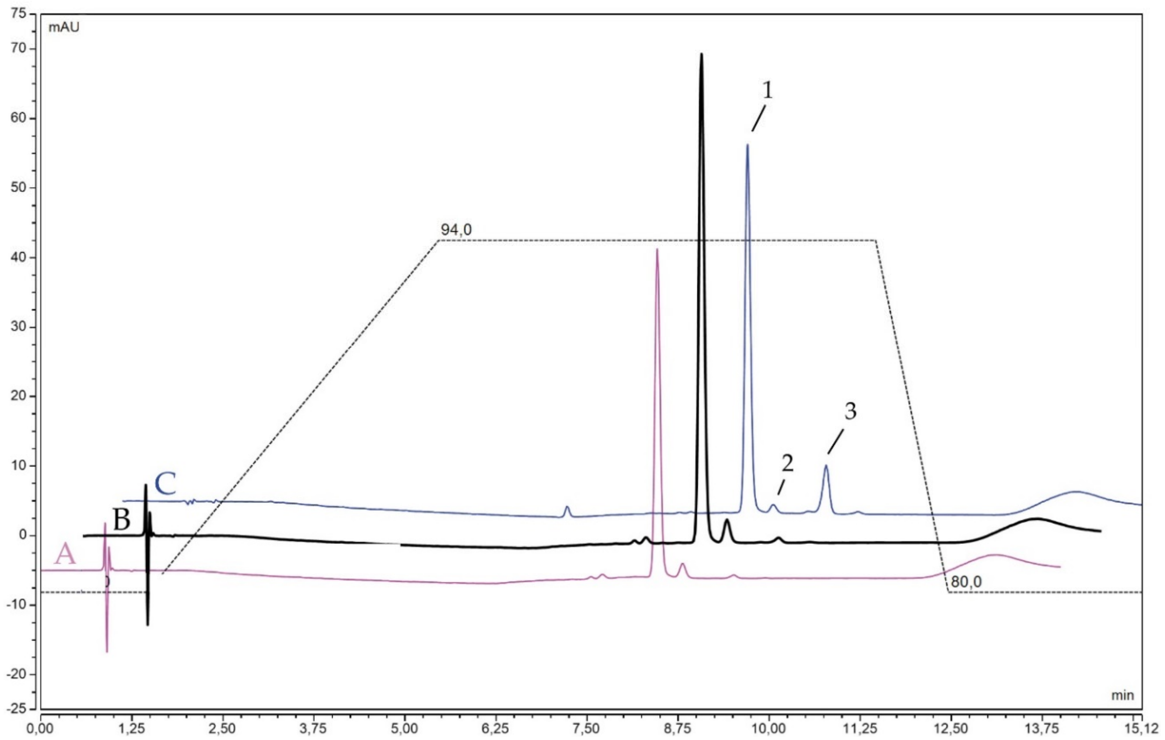

Figure S2. HPLC-DAD chromatograms of bulk oil, powder and micellated formulation of lutein + zeaxanthin. Detection wavelength was set to 445 nm, bulk oil and micellated lutein were subjected to saponification prior to analysis. Bulk oil was then diluted in acetonitrile to 18 µg/mL (A), powder to 80 µg/mL (B) and micellated lutein+zeaxanthin to 650 µg/mL (C). Peak 1 (R_t = 8.58 min, absorption maxima at 445 and 472 nm) represents (all-E) lutein, peak 2 (R_t = 8.93 min, absorption maxima at 449 and 476 nm) represents meso-zeaxanthin and peak 3 (R_t = 9.66 min, absorption maxima at 440 and 467 nm) represents (13-Z) lutein.

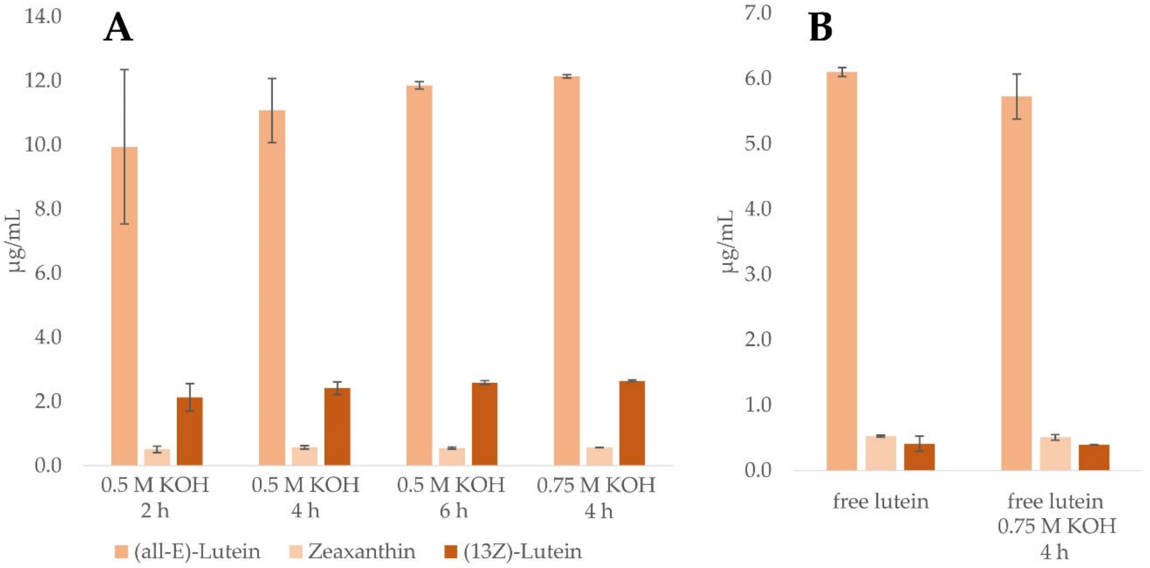

Figure S3. Optimization of lutein ester saponification conditions. Results of HPLC-DAD measurements of micellated lutein ester (1.4 mg/mL) after saponification with 0.5 M KOH for 2, 4 and 6 h as well as with 0.75 M KOH for 4 h (A) and a control experiment using 6 µg/mL of free lutein without saponification step and after incubation at the optimized conditions with 0.75 M KOH for 4 h (B) (n=3 per condition).

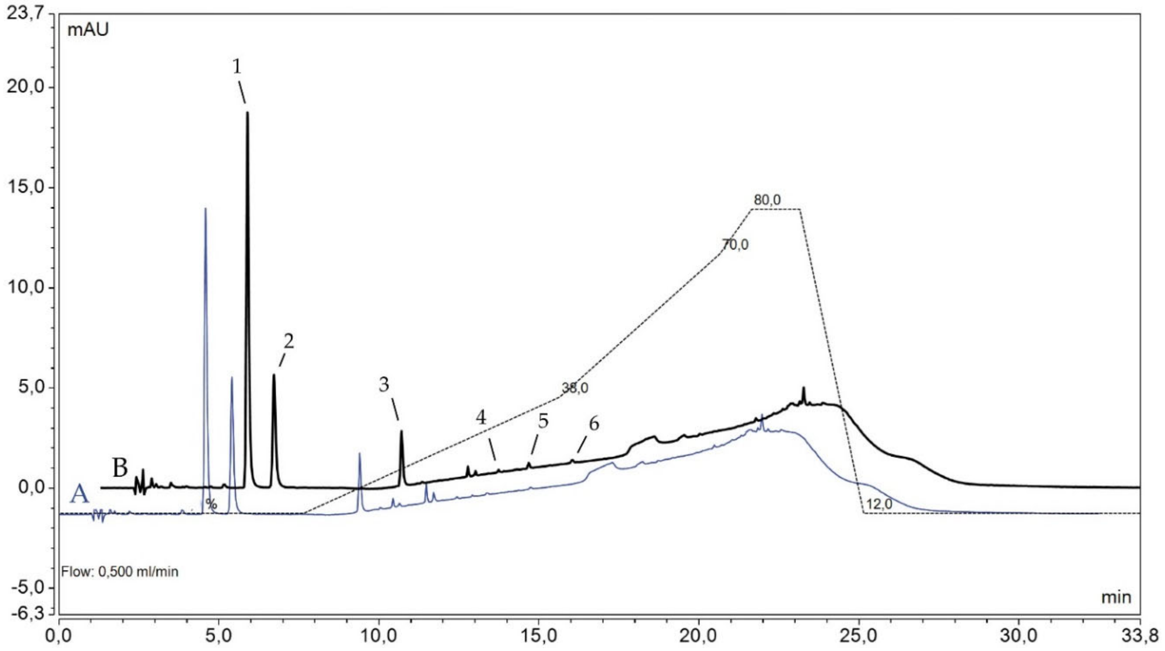

Figure S4. HPLC-DAD chromatograms of micellated and powder formulation of soy isoflavones. Detection wavelength was set to 260 nm, powder formulation was diluted in acetonitrile to 20 µg/mL (A) and micellated isoflavones to 600 µg/mL (B). Peak 1 (R_t = 4.58 min, absorption maxima at 250 and 295 nm) represents daidzin, peak 2 (R_t = 5.41 min, absorption maxima at 258 and 320 nm) represents glycitin and peak 3 (R_t = 9.40 min, absorption maxima at 259 and 327 nm) represents genistin, while peaks 4 (R_t = 12.43 min), 5 (R_t = 12.92 min) and 6 (R_t = 14.36 min) represent the aglycones daidzein, glycitein and genistein.

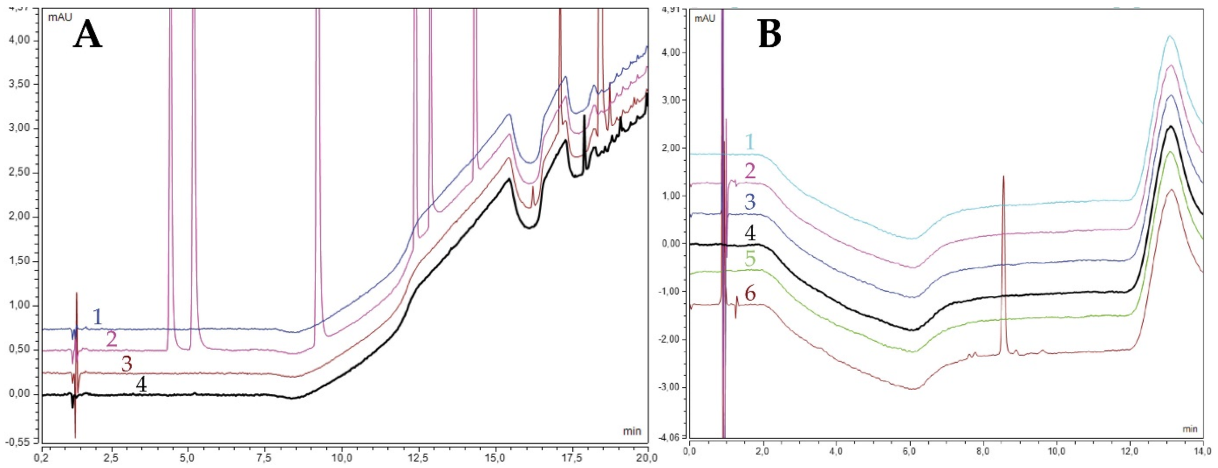

Figure S5. HPLC-DAD chromatograms of the bioenhancer extracts measured with the isoflavone and lutein method, respectively. For determination of potential isoflavone content (A), a blank injection is shown in chromatogram 1, a mixture of the six isoflavone standards at a concentration of 5 µg/mL in chromatogram 2, followed by BPE at a concentration of 100 µg/mL in chromatogram 3 and GiE at 1 mg/mL in chromatogram 4. Similarly, potential lutein content (B) was determined. A blank injection is shown in chromatogram 1, followed by BPE at 100 µg/mL in chromatogram 2, GiE at 1 mg/mL in chromatogram 3, the same extracts after saponification at the conditions described for lutein extraction in chromatograms 4 and 5, and finally a lutein standard at 1 µg/mL in chromatogram 6.

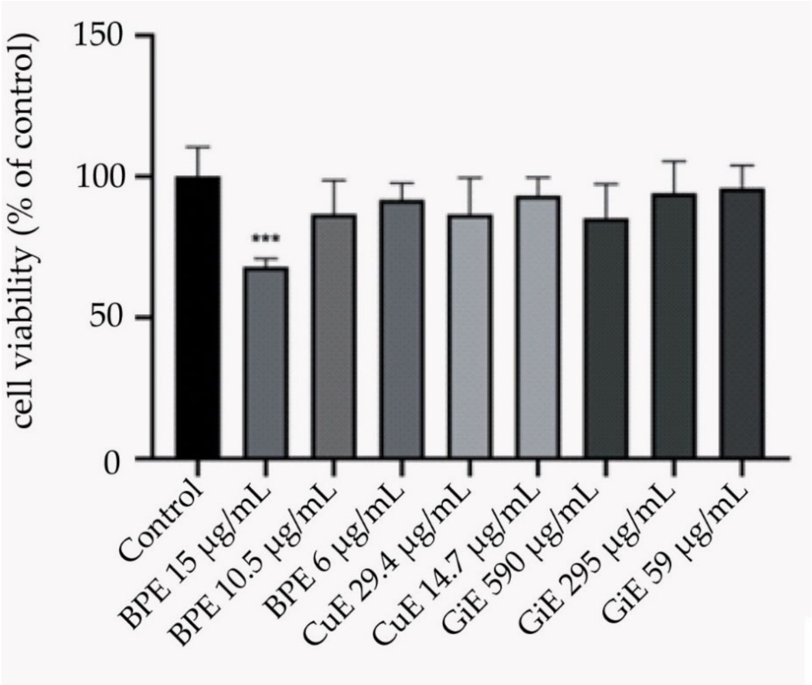

Figure S6. Results of cell viability assays of the bioenhancer extracts. CaCo-2 cells were treated either with pure FaSSIF medium or the bioenhancer extracts (BPE = black pepper extract, CuE = curcuma exract, GiE = ginger extract) in the indicated concentrations for 4h. Cell viability is given as percent of control, error bars represent mean +/- SD (n=4). Statistically significant differences are denoted as* ($p \leqslant 0.05$), ** ($\leqslant 0.01$) and *** ($\leqslant 0.001$).

Table S1. Raw data for uptake experiments of curcuma formulations in differentiated CaCo-2 cells. Normalized cellular uptake expressed as µg curcuminoids per mg cellular protein, determined by HPLC-FLD and Bradford assay, respectively. Relative cellular uptake is calculated with respect to the regular curcumin formulation within each experiment.

Condition	Normalized cellular uptake (µg/mg protein)				Relative cellular uptake
	Bisdemethoxycurcumin	Demethoxycurcumin	Curcumin	Sum curcuminoids	Sum curcuminoids
CUR_REG_Exp1_Rep1	0.0029	0.0048	0.0622	0.0699	0.960
CUR_REG_Exp1_Rep2	0.0026	0.0038	0.0631	0.0696	0.956
CUR_REG_Exp1_Rep3	0.0028	0.0042	0.0719	0.0789	1.084
CUR_CD_Exp1_Rep1	0.0044	0.0092	0.1843	0.1979	2.720
CUR_CD_Exp1_Rep2	0.0034	0.0054	0.1240	0.1328	1.825
CUR_CD_Exp1_Rep3	0.0050	0.0118	0.1943	0.2112	2.902
CUR_REG_Exp2_Rep1	0.0030	0.0006	0.0193	0.0229	0.936
CUR_REG_Exp2_Rep2	0.0027	n.a.	0.0157	0.0184	0.750
CUR_REG_Exp2_Rep3	0.0037	0.0001	0.0284	0.0323	1.315
CUR_CD_Exp2_Rep1	0.0039	0.0057	0.0743	0.0838	3.416

CUR_CD_Exp2_Rep2	0.0047	0.0022	0.0436	0.0505	2.058
CUR_CD_Exp2_Rep3	0.0042	n.a.	0.0247	0.0290	1.181

Table S2. Raw data for uptake experiments of CD-complexed curcuma with bioenhancer extracts in differentiated CaCo-2 cells. Normalized cellular uptake expressed as μg curcuminoids per mg cellular protein, determined by HPLC-FLD and Bradford assay, respectively. Relative cellular uptake was calculated with respect to the CD-complexed curcuma extract within each experiment.

Condition	Normalized cellular uptake (μg/mg protein)				Relative cellular uptake
	Bisdemeth-oxycurcu-min	Demethox-ycurcumin	Curcumin	Sum curcu-minoids	Sum curcu-minoids
CUR_CD_Exp1_Rep1	0.0044	0.0092	0.1843	0.1979	1.096
CUR_CD_Exp1_Rep2	0.0034	0.0054	0.1240	0.1328	0.735
CUR_CD_Exp1_Rep3	0.0050	0.0118	0.1943	0.2112	1.169
CUR_CD+BPE_Exp1_Rep1	0.0089	0.0381	0.4821	0.5291	2.929
CUR_CD+BPE _Exp1_Rep2	0.0094	0.0386	0.4940	0.5420	3.001
CUR_CD+BPE _Exp1_Rep3	0.0060	0.0277	0.3610	0.3947	2.185
CUR_CD_Exp2_Rep1	0.0039	0.0057	0.0743	0.0838	3.416
CUR_CD_Exp2_Rep2	0.0047	0.0022	0.0436	0.0505	2.058
CUR_CD_Exp2_Rep3	0.0042	n.a.	0.0247	0.0290	1.181
CUR_CD+BPE+GiE_Exp2_Rep1	0.00614	0.00069	0.06289	0.06973	1.8326
CUR_CD+BPE+GiE_Exp2_Rep2	0.00658	0.00111	0.07589	0.08358	2.1966
CUR_CD+BPE+GiE_Exp2_Rep3	0.00632	0.00025	0.05355	0.06012	1.5800
CUR_CD_Exp3_Rep1	0.0016	0.0018	0.0359	0.0393	0.748
CUR_CD_Exp3_Rep2	0.0020	0.0032	0.0608	0.0660	1.256
CUR_CD_Exp3_Rep3	0.0021	0.0025	0.0477	0.0523	0.996
CUR_CD+BPE_Exp3_Rep1	0.0025	0.0076	0.1340	0.1440	2.742
CUR_CD+BPE_Exp3_Rep2	0.0032	0.0097	0.1873	0.2001	3.810
CUR_CD+BP _Exp3_Rep3	0.0023	0.0073	0.1406	0.1503	2.861
CUR_CD_Exp4_Rep1	0.00149	0.00603	0.26413	0.27164	0.822
CUR_CD_Exp4_Rep2	0.00184	0.00576	0.32308	0.33067	1.001
CUR_CD_Exp4_Rep3	0.00223	0.01329	0.37328	0.38879	1.177
CUR_CD+BPE_Exp4_Rep1	0.00241	0.01310	0.60907	0.62458	1.891
CUR_CD+BPE_Exp4_Rep2	0.00259	0.01134	0.52645	0.54037	1.636

CUR_CD+BPE_Exp4_Rep3	0.00168	0.01053	0.41855	0.43076	1.304
CUR_CD_Exp5_Rep1	0.00142	0.00479	0.24656	0.25277	1.196
CUR_CD_Exp5_Rep2	0.00139	0.00463	0.19187	0.19789	0.936
CUR_CD_Exp5_Rep3	0.00145	0.00552	0.17642	0.18339	0.868
CUR_CD+BPE_Exp5_Rep1	0.00243	0.01278	0.49360	0.50881	2.407
CUR_CD+BPE_Exp5_Rep2	0.00189	0.01233	0.39294	0.40715	1.926
CUR_CD+BPE_Exp5_Rep3	0.00279	0.01625	0.47888	0.49791	2.356
CUR_CD_Exp6_Rep1	0.00264	0.00157	0.06552	0.06973	1.015
CUR_CD_Exp6_Rep2	0.00241	0.00081	0.05737	0.06060	0.882
CUR_CD_Exp6_Rep3	0.00243	0.00097	0.07241	0.07581	1.103
CUR_CD+BPE_Exp6_Rep1	0.00247	0.00295	0.08221	0.08763	1.275
CUR_CD+BPE_Exp6_Rep2	0.00283	0.00298	0.10226	0.10806	1.573
CUR_CD+BPE_Exp6_Rep3	0.00248	0.00270	0.09560	0.10078	1.467
CUR_CD+BPE+GiE_Exp6_Rep1	0.0028	0.0029	0.0837	0.0894	1.3010
CUR_CD+BPE+GiE_Exp6_Rep2	0.0031	0.0018	0.1156	0.1205	1.7541
CUR_CD+BPE+GiE_Exp6_Rep3	0.0030	0.0042	0.1011	0.1083	1.5765

Table S3. Raw data for uptake experiments of lutein formulations in undifferentiated CaCo-2 cells. Normalized cellular uptake expressed as μg xantophylls per mg cellular protein, determined by HPLC-DAD and Bradford assay, respectively.

Condition	Normalized cellular uptake (μg/mg protein)			
	(all-E)-Lutein	Meso-Zeaxan-thin	(13Z)-Lutein	total xantopylls
LUT_Oil_Exp1_Rep1	0.3868	0.0209	0.0804	0.4881
LUT_Oil_Exp1_Rep2	0.3709	0.0212	0.0672	0.4593
LUT_Oil_Exp1_Rep3	0.4118	0.0235	0.0645	0.4997
LUT_Mic_Exp1_Rep1	0.3504	0.0152	0.0955	0.4611
LUT_Mic_Exp1_Rep2	0.2604	0.0119	0.0684	0.3407
LUT_Mic_Exp1_Rep3	0.3936	0.0181	0.0883	0.5000
LUT_Pow_Exp1_Rep1	1.909	0.155	0.205	2.269
LUT_Pow_Exp1_Rep2	2.011	0.169	0.205	2.384
LUT_Pow_Exp1_Rep3	2.091	0.170	0.191	2.452
LUT_Oil_Exp2_Rep1	0.2830	0.0268	0.0890	0.3988

LUT_Oil_Exp2_Rep2	0.3738	0.0303	0.0938	0.4979
LUT_Oil_Exp2_Rep3	0.4042	0.0311	0.1152	0.5506
LUT_Mic_Exp2_Rep1	0.3346	0.0134	0.0960	0.4440
LUT_Mic_Exp2_Rep2	0.2773	0.0151	0.0851	0.3776
LUT_Mic_Exp2_Rep3	0.2055	0.0125	0.0625	0.2806
LUT_Pow_Exp2_Rep1	1.5244	0.1220	0.2557	1.9021
LUT_Pow_Exp2_Rep2	1.8921	0.1978	0.1478	2.2376
LUT_Pow_Exp2_Rep3	1.9286	0.1574	0.1691	2.2552

Table S4. Raw data for uptake experiments of lutein formulations with bioenhancer extracts in undifferentiated CaCo-2 cells. Normalized cellular uptake expressed as µg xantophylls per mg cellular protein, determined by HPLC-DAD and Bradford assay, respectively. Relative cellular uptake was calculated with respect to the lutein condition without bioenhancer extracts within each experiment.

Condition	Normalized cellular uptake (µg/mg protein)				Relative cellular uptake
	(all-E)-Lutein	Meso-Zeaxanthin	(13Z)-Lutein	total xantopylls	total xantopylls
LUT_Oil_Exp1_Rep1	0.38681	0.02089	0.08043	0.4881	1.012
LUT_Oil_Exp1_Rep2	0.37092	0.02124	0.06718	0.4593	0.952
LUT_Oil_Exp1_Rep3	0.41179	0.02349	0.06446	0.4997	1.036
LUT_Oil+CuE_Exp1_Rep1	0.3873	0.0217	0.0735	0.4824	1.000
LUT_Oil+CuE_Exp1_Rep2	0.3291	0.0206	0.0724	0.4222	0.875
LUT_Oil+CuE_Exp1_Rep3	0.3331	0.0199	0.0588	0.4118	0.854
LUT_Oil+BPE_Exp1_Rep1	0.2459	0.0148	0.0470	0.3077	0.638
LUT_Oil+BPE_Exp1_Rep2	0.4506	0.0249	0.0711	0.5466	1.133
LUT_Oil+BPE_Exp1_Rep3	0.3531	0.0218	0.0617	0.4366	0.905
LUT_Oil+GiE_Exp1_Rep1	0.3824	0.0190	0.0571	0.4585	0.939
LUT_Oil+GiE_Exp1_Rep2	0.4461	0.0243	0.0699	0.5403	1.107
LUT_Oil+GiE_Exp1_Rep3	0.3531	0.0204	0.0616	0.4351	0.891
LUT_Mic_Exp1_Rep1	0.3504	0.0152	0.0955	0.4776	1.068
LUT_Mic_Exp1_Rep2	0.2604	0.0119	0.0684	0.3482	0.779
LUT_Mic_Exp1_Rep3	0.3936	0.0181	0.0883	0.5153	1.153
LUT_Mic+CuE_Exp1_Rep1	0.4355	0.0192	0.0942	0.5584	1.169
LUT_Mic+CuE_Exp1_Rep2	0.2911	0.0126	0.0700	0.3808	0.797
LUT_Mic+CuE_Exp1_Rep3	0.2888	0.0130	0.0710	0.3858	0.808

LUT_Mic+BPE_Exp1_Rep1	0.3542	0.0154	0.0949	0.4806	1.075
LUT_Mic+BPE_Exp1_Rep2	0.4623	0.0200	0.1068	0.6077	1.359
LUT_Mic+BPE_Exp1_Rep3	0.3700	0.0157	0.0892	0.4899	1.096
LUT_Mic+GiE_Exp1_Rep1	0.3641	0.0167	0.0818	0.4782	1.070
LUT_Mic+GiE_Exp1_Rep2	0.2455	0.0118	0.0547	0.3182	0.712
LUT_Mic+GiE_Exp1_Rep3	0.3419	0.0153	0.0753	0.4474	1.001
LUT_Pow_Exp1_Rep1	1.9089	0.1553	0.2051	2.2693	0.958
LUT_Pow_Exp1_Rep2	2.0105	0.1693	0.2047	2.3845	1.007
LUT_Pow_Exp1_Rep3	2.0905	0.1704	0.1908	2.4517	1.035
LUT_Pow+CuE_Exp1_Rep1	2.014	0.165	0.222	2.401	1.014
LUT_Pow+CuE_Exp1_Rep2	1.803	0.144	0.223	2.170	0.916
LUT_Pow+CuE_Exp1_Rep3	1.890	0.148	0.196	2.235	0.944
LUT_Pow+BPE_Exp1_Rep1	2.475	0.202	0.321	2.174	0.918
LUT_Pow+BPE_Exp1_Rep2	1.797	0.147	0.244	2.188	0.924
LUT_Pow+BPE_Exp1_Rep3	2.635	0.206	0.303	2.004	0.846
LUT_Pow+GiE_Exp1_Rep1	1.770	0.144	0.197	2.111	0.891
LUT_Pow+GiE_Exp1_Rep2	1.802	0.143	0.201	2.145	0.906
LUT_Pow+GiE_Exp1_Rep3	1.454	0.118	0.157	1.729	0.730

Table S5. Raw data for uptake experiments of lutein formulations with bioenhancer extracts in differentiated CaCo-2 cells. Normalized cellular uptake expressed as µg xantophylls per mg cellular protein, determined by HPLC-DAD and Bradford assay, respectively. Relative cellular uptake was calculated with respect to the lutein condition without bioenhancer extracts within each experiment.

Condition	Normalized cellular uptake (µg/mg protein)				Relative cellular uptake
	(all-E)-Lutein	Meso-Zeaxanthin	(13Z)-Lutein	total xantopylls	total xantopylls
LUT_Pow_Exp1_Rep1	1.073	1.128	1.037	3.238	1.070
LUT_Pow_Exp1_Rep2	0.925	1.004	0.874	2.803	0.920
LUT_Pow_Exp1_Rep3	1.002	0.868	1.089	2.959	1.010
LUT_Pow+CuE_Exp1_Rep1	1.694	1.702	1.286	4.681	1.566
LUT_Pow+CuE_Exp1_Rep2	1.217	1.282	0.962	3.461	1.137
LUT_Pow+CuE_Exp1_Rep3	2.210	2.044	1.920	6.174	2.104
LUT_Pow+BPE_Exp1_Rep1	0.897	n.a.	0.817	1.714	0.822
LUT_Pow+BPE_Exp1_Rep2	1.141	1.278	1.087	3.506	1.125

LUT_Pow+BPE_Exp1_Rep3	1.143	1.176	0.998	3.317	1.098
LUT_Pow+GiE_Exp1_Rep1	1.453	1.474	1.212	4.139	1.363
LUT_Pow+GiE_Exp1_Rep2	1.463	1.274	1.584	4.322	1.463
LUT_Pow+GiE_Exp1_Rep3	1.172	1.054	1.402	3.627	1.213
LUT_Pow_Exp2_Rep1	1.109	n.a.	1.133	2.243	1.118
LUT_Pow_Exp2_Rep2	1.071	n.a.	1.046	2.117	1.061
LUT_Pow_Exp2_Rep3	0.820	n.a.	0.821	1.641	0.820
LUT_Pow+CuE_Exp2_Rep1	1.465	n.a.	1.210	2.675	1.370
LUT_Pow+CuE_Exp2_Rep2	1.527	n.a.	1.245	2.772	1.421
LUT_Pow+CuE_Exp2_Rep3	1.458	n.a.	1.424	2.882	1.445
LUT_Pow+GiE_Exp2_Rep1	1.095	n.a.	1.039	2.135	1.074
LUT_Pow+GiE_Exp2_Rep2	1.210	n.a.	1.158	2.368	1.190
LUT_Pow+GiE_Exp2_Rep3	0.831	n.a.	0.938	1.769	0.871
LUT_Pow_Exp3_Rep1	0.984	1.116	0.923	3.024	0.960
LUT_Pow_Exp3_Rep2	1.056	0.928	1.078	3.061	1.060
LUT_Pow_Exp3_Rep3	0.960	0.957	0.998	2.915	0.980
LUT_Pow+CuE_Exp3_Rep1	0.942	0.550	0.927	2.419	0.910
LUT_Pow+CuE_Exp3_Rep2	1.258	0.939	1.314	3.511	1.258
LUT_Pow+CuE_Exp3_Rep3	1.177	1.234	1.179	3.589	1.171
LUT_Pow+GiE_Exp3_Rep1	0.793	0.573	0.821	2.186	0.790
LUT_Pow+GiE_Exp3_Rep2	0.823	0.900	0.901	2.625	0.857
LUT_Pow+GiE_Exp3_Rep3	0.399	0.356	0.471	1.226	0.429
LUT_Pow_Exp4_Rep1	1.191	0.095	0.054	1.341	0.894
LUT_Pow_Exp4_Rep2	1.425	0.123	0.068	1.617	1.078
LUT_Pow_Exp4_Rep3	1.356	0.111	0.076	1.543	1.028
LUT_Pow+CD_Exp4_Rep1	1.652	0.130	0.085	1.867	1.245
LUT_Pow+CD_Exp4_Rep2	1.135	0.096	0.064	1.295	0.863
LUT_Pow+CD_Exp4_Rep3	1.198	0.099	0.106	1.404	0.936
LUT_Pow+CuE+BPE_Exp4_Rep1	1.570	0.164	0.127	1.861	1.241
LUT_Pow+CuE+BPE_Exp4_Rep2	1.992	0.086	0.114	2.191	1.461
LUT_Pow+CuE+BPE_Exp4_Rep3	1.733	0.100	n.a.	1.834	1.222
LUT_Pow+CuE+BPE+GiE_Exp4_Rep1	1.248	0.121	0.288	1.657	1.105
LUT_Pow+CuE+BPE+GiE_Exp4_Rep2	2.180	0.418	0.301	2.899	1.933
LUT_Pow+CuE+BPE+GiE_Exp4_Rep3	1.942	n.a.	0.137	2.079	1.386

Table S6. Raw data for transport experiments of isoflavone formulations in differentiated CaCo-2 cells. Apparent permeability coefficient (Papp) in cm/s, based on concentrations in the apical compartment the start of the experiments and the basolateral compartment after 4 h incubation, determined by HPLC-DAD.

Condition	Apparent permeability coefficient P_{app} (cm/s)			
	Daidzein	Glycitein	Genistein	Total isoflavones
IF_Pow_Exp1_Rep1	3.76E-06	2.92E-06	1.52E-06	3.22E-06
IF_Pow_Exp1_Rep2	3.91E-06	3.18E-06	1.62E-06	3.41E-06
IF_Pow_Exp1_Rep3	3.83E-06	2.88E-06	1.36E-06	3.23E-06
IF_Mic_Exp1_Rep1	4.15E-06	3.01E-06	1.78E-06	3.49E-06
IF_Mic_Exp1_Rep2	3.64E-06	2.52E-06	1.47E-06	3.02E-06
IF_Mic_Exp1_Rep3	4.18E-06	3.04E-06	1.76E-06	3.51E-06
IF_Pow_Exp2_Rep1	4.30E-06	4.44E-06	3.15E-06	4.25E-06
IF_Pow_Exp2_Rep2	4.25E-06	3.87E-06	2.51E-06	3.93E-06
IF_Pow_Exp2_Rep3	4.35E-06	4.14E-06	2.70E-06	4.11E-06
IF_Mic_Exp2_Rep1	4.51E-06	4.48E-06	2.65E-06	4.27E-06
IF_Mic_Exp2_Rep2	4.56E-06	4.44E-06	2.52E-06	4.26E-06
IF_Mic_Exp2_Rep3	4.61E-06	4.76E-06	2.90E-06	4.44E-06

Table S7. Raw data of transport experiments of isoflavone formulations with bioenhancer extracts in differentiated CaCo-2 cells. Apparent permeability coefficient (Papp) in cm/s, based on concentrations in the apical compartment the start of the experiments and the basolateral compartment after 4 h incubation, determined by HPLC-DAD.

Condition	Apparent permeability coefficient P_{app} (cm/s)			
	Daidzein	Glycitein	Genistein	Total isoflavones
IF_Pow_Exp1_Rep1	3.76E-06	2.92E-06	1.52E-06	3.22E-06
IF_Pow_Exp1_Rep2	3.91E-06	3.18E-06	1.62E-06	3.41E-06
IF_Pow_Exp1_Rep3	3.83E-06	2.88E-06	1.36E-06	3.23E-06
IF_Pow+BPE_Exp1_Rep1	3.75E-06	2.98E-06	1.49E-06	3.24E-06
IF_Pow+BPE_Exp1_Rep2	3.93E-06	3.15E-06	1.57E-06	3.41E-06
IF_Pow+BPE_Exp1_Rep3	4.38E-06	3.79E-06	1.91E-06	3.93E-06

IF_Pow+CuE_Exp1_Rep1	4.18E-06	3.67E-06	1.87E-06	3.77E-06
IF_Pow+CuE_Exp1_Rep2	4.17E-06	3.76E-06	2.13E-06	3.83E-06
IF_Pow+CuE_Exp1_Rep3	4.67E-06	4.15E-06	2.31E-06	4.25E-06
IF_Pow+GiE_Exp1_Rep1	4.11E-06	3.72E-06	1.93E-06	3.76E-06
IF_Pow+GiE_Exp1_Rep2	4.20E-06	3.45E-06	1.67E-06	3.67E-06
IF_Pow+GiE_Exp1_Rep3	4.51E-06	3.72E-06	1.74E-06	3.95E-06
IF_Mic_Exp1_Rep1	4.15E-06	3.01E-06	1.78E-06	3.49E-06
IF_Mic_Exp1_Rep2	3.64E-06	2.52E-06	1.47E-06	3.02E-06
IF_Mic_Exp1_Rep3	4.18E-06	3.04E-06	1.76E-06	3.51E-06
IF_Mic+BPE_Exp1_Rep1	4.35E-06	3.35E-06	1.89E-06	3.72E-06
IF_Mic+BPE_Exp1_Rep2	4.06E-06	3.18E-06	1.89E-06	3.51E-06
IF_Mic+BPE_Exp1_Rep3	3.78E-06	3.10E-06	1.73E-06	3.31E-06
IF_Mic+CuE_Exp1_Rep1	4.29E-06	3.04E-06	1.87E-06	3.59E-06
IF_Mic+CuE_Exp1_Rep2	4.67E-06	3.71E-06	2.24E-06	4.06E-06
IF_Mic+CuE_Exp1_Rep3	4.37E-06	3.51E-06	2.20E-06	3.82E-06
IF_Mic+GiE_Exp1_Rep1	4.77E-06	3.49E-06	1.96E-06	4.01E-06
IF_Mic+GiE_Exp1_Rep2	4.70E-06	3.30E-06	1.88E-06	3.90E-06
IF_Mic+GiE_Exp1_Rep3	5.16E-06	3.99E-06	2.09E-06	4.40E-06
IF_Pow_Exp2_Rep1	4.30E-06	4.44E-06	3.15E-06	4.25E-06
IF_Pow_Exp2_Rep2	4.25E-06	3.87E-06	2.51E-06	3.93E-06
IF_Pow_Exp2_Rep3	4.35E-06	4.14E-06	2.70E-06	4.11E-06
IF_Pow+CuE_Exp2_Rep1	4.64E-06	4.67E-06	3.48E-06	4.54E-06
IF_Pow+CuE_Exp2_Rep2	4.73E-06	4.75E-06	3.32E-06	4.61E-06
IF_Pow+CuE_Exp2_Rep3	4.91E-06	4.99E-06	3.39E-06	4.80E-06
IF_Pow+GiE_Exp2_Rep1	5.01E-06	4.86E-06	3.04E-06	4.76E-06
IF_Pow+GiE_Exp2_Rep2	4.66E-06	4.68E-06	3.01E-06	4.51E-06
IF_Pow+GiE_Exp2_Rep3	4.97E-06	4.89E-06	3.07E-06	4.76E-06
IF_Mic_Exp2_Rep1	4.51E-06	4.48E-06	2.65E-06	4.27E-06
IF_Mic_Exp2_Rep2	4.56E-06	4.44E-06	2.52E-06	4.26E-06
IF_Mic_Exp2_Rep3	4.61E-06	4.76E-06	2.90E-06	4.44E-06
IF_Mic+CuE_Exp2_Rep1	4.79E-06	5.00E-06	3.09E-06	4.65E-06
IF_Mic+CuE_Exp2_Rep2	4.53E-06	4.58E-06	2.89E-06	4.34E-06
IF_Mic+GiE_Exp2_Rep1	5.16E-06	5.35E-06	3.21E-06	4.98E-06
IF_Mic+GiE_Exp2_Rep2	5.03E-06	5.01E-06	2.93E-06	4.76E-06
IF_Mic+GiE_Exp2_Rep3	4.76E-06	4.84E-06	3.14E-06	4.58E-06

IF_Pow_Exp3_Rep1	5.02E-06	5.11E-06	3.16E-06	4.88E-06
IF_Pow_Exp3_Rep2	5.10E-06	5.24E-06	3.29E-06	4.98E-06
IF_Pow_Exp3_Rep3	5.27E-06	5.51E-06	3.60E-06	5.21E-06
IF_Pow+CuE+BPE+GiE+_Exp3_Rep1	5.36E-06	5.45E-06	3.68E-06	5.24E-06
IF_Pow+CuE+BPE+GiE+_Exp3_Rep2	5.56E-06	5.66E-06	3.62E-06	5.42E-06
IF_Pow+CuE+BPE+GiE+_Exp3_Rep3	5.71E-06	6.10E-06	4.10E-06	5.71E-06
IF_Pow_Exp4_Rep1	4.81E-06	3.71E-06	2.68E-06	4.19E-06
IF_Pow_Exp4_Rep2	4.86E-06	3.64E-06	2.77E-06	4.19E-06
IF_Pow_Exp4_Rep3	4.15E-06	3.38E-06	2.55E-06	3.71E-06
IF_Pow+CuE+BPE+GiE+_Exp4_Rep1	5.12E-06	4.02E-06	3.23E-06	4.51E-06
IF_Pow+CuE+BPE+GiE+_Exp4_Rep2	5.13E-06	3.92E-06	3.08E-06	4.47E-06
IF_Pow+CuE+BPE+GiE+_Exp4_Rep3	6.22E-06	4.71E-06	3.85E-06	5.41E-06

Table S8. Raw data of metabolite concentrations in transport experiments of isoflavones with bio-enhancer extracts in differentiated CaCo-2 cells. Concentration of metabolites was determined as change in the concentration of the aglycones after β-glucuronidase digestion. Determined in apical and basolateral compartments after 4 h incubation by HPLC-DAD.

Condition	Concentration (µM)			
	Daidzein metabolites	Glycitein metabolites	Genistein metabolites	Sum metabolites
IF_Pow_Exp1_Rep1_apical	0.0947	0.1908	0.0985	0.3840
IF_Pow_Exp1_Rep2_apical	0.0843	0.1692	0.0912	0.3447
IF_Pow_Exp1_Rep3_apical	0.1034	0.2116	0.1092	0.4243
IF_Pow_Exp1_Rep1_basolateral	0.043	0.078	0.019	0.140
IF_Pow_Exp1_Rep2_basolateral	0.039	0.076	0.022	0.137
IF_Pow_Exp1_Rep3_basolateral	0.036	0.101	0.031	0.168
IF_Pow+CuE_Exp1_Rep1_apical	0.0566	0.1315	0.0734	0.2615
IF_Pow+CuE_Exp1_Rep2_apical	0.0508	0.1427	0.0806	0.2741
IF_Pow+CuE_Exp1_Rep3_apical	0.0513	0.1233	0.0767	0.2514
IF_Pow+CuE_Exp1_Rep1_basolateral	0.0297	0.0579	0.0274	0.1150
IF_Pow+CuE_Exp1_Rep2_basolateral	0.0062	0.0736	0.0243	0.1042
IF_Pow+CuE_Exp1_Rep3_basolateral	0.0508	0.1427	0.0806	0.2741
IF_Pow+BPE_Exp1_Rep1_apical	0.1157	0.1833	0.0989	0.3979
IF_Pow+BPE_Exp1_Rep2_apical	0.0873	0.1711	0.0985	0.3570
IF_Pow+BPE_Exp1_Rep3_apical	0.0905	0.1746	0.0978	0.3630

IF_Pow+BPE_Exp1_Rep1_basolateral	0.0471	0.0900	0.0267	0.1638
IF_Pow+BPE_Exp1_Rep2_basolateral	0.0449	0.0853	0.0241	0.1544
IF_Pow+BPE_Exp1_Rep3_basolateral	0.0180	0.0713	0.0243	0.1137
IF_Pow+GiE_Exp1_Rep1_apical	0.0595	0.1378	0.0788	0.2761
IF_Pow+GiE_Exp1_Rep2_apical	0.0779	0.1821	0.0933	0.3533
IF_Pow+GiE_Exp1_Rep3_apical	0.1066	0.2172	0.1111	0.4349
IF_Pow+GiE_Exp1_Rep1_basolateral	0.0137	0.0642	0.0200	0.0979
IF_Pow+GiE_Exp1_Rep2_basolateral	0.0396	0.0820	0.0245	0.1462
IF_Pow+GiE_Exp1_Rep3_basolateral	0.0424	0.0870	0.0243	0.1537
IF_Mic_Exp2_Rep1_apical	0.1580	0.1880	0.1705	0.5165
IF_Mic_Exp2_Rep2_apical	0.2316	0.2470	0.2065	0.6850
IF_Mic_Exp2_Rep3_apical	0.1661	0.1985	0.1750	0.5396
IF_Mic_Exp2_Rep1_basolateral	0.0983	0.1250	0.0737	0.2971
IF_Mic_Exp2_Rep2_basolateral	0.1152	0.1231	0.0707	0.3090
IF_Mic_Exp2_Rep3_basolateral	0.0895	0.1129	0.0714	0.2738
IF_Mic+CuE_Exp2_Rep1_apical	0.1673	0.2181	0.1915	0.5769
IF_Mic+CuE_Exp2_Rep2_apical	0.1200	0.1693	0.1504	0.4397
IF_Mic+CuE_Exp2_Rep3_apical	0.1464	0.1640	0.1498	0.4603
IF_Mic+CuE_Exp2_Rep1_basolateral	0.1023	0.1379	0.0749	0.3151
IF_Mic+CuE_Exp2_Rep2_basolateral	0.0842	0.1130	0.0779	0.2751
IF_Mic+CuE_Exp2_Rep3_basolateral	0.0845	0.1120	0.0799	0.2764
IF_Mic+BPE_Exp2_Rep1_apical	0.1725	0.1941	0.1699	0.5365
IF_Mic+BPE_Exp2_Rep2_apical	0.1664	0.1810	0.1627	0.5101
IF_Mic+BPE_Exp2_Rep3_apical	0.1816	0.2006	0.2115	0.5936
IF_Mic+BPE_Exp2_Rep1_basolateral	0.1188	0.1227	0.0777	0.3192
IF_Mic+BPE_Exp2_Rep2_basolateral	0.0920	0.0997	0.0688	0.2605
IF_Mic+BPE_Exp2_Rep3_basolateral	0.0882	0.0880	0.0772	0.2533
IF_Mic+GiE_Exp2_Rep1_apical	0.1465	0.1682	0.1436	0.4583
IF_Mic+GiE_Exp2_Rep2_apical	0.2015	0.2162	0.1731	0.5907
IF_Mic+GiE_Exp2_Rep3_apical	0.1705	0.2130	0.1818	0.5653
IF_Mic+GiE_Exp2_Rep1_basolateral	0.1046	0.1469	0.0819	0.3334
IF_Mic+GiE_Exp2_Rep2_basolateral	0.1402	0.1779	0.1031	0.4213
IF_Mic+GiE_Exp2_Rep3_basolateral	0.1146	0.1738	0.0941	0.3824
